# Multi‐Omics Analysis Reveals the Mechanism Underlying the Edaphic Adaptation in Wild Barley at Evolution Slope (Tabigha)

**DOI:** 10.1002/advs.202101374

**Published:** 2021-08-13

**Authors:** Shengguan Cai, Qiufang Shen, Yuqing Huang, Zhigang Han, Dezhi Wu, Zhong‐Hua Chen, Eviatar Nevo, Guoping Zhang

**Affiliations:** ^1^ College of Agriculture and Biotechnology Zhejiang University Hangzhou 310058 China; ^2^ Institute of Crop Science Hangzhou Academy of Agricultural Sciences Hangzhou 310024 China; ^3^ School of Science Western Sydney University Penrith NSW 2751 Australia; ^4^ Hawkesbury Institute for the Environment Western Sydney University Penrith NSW 2751 Australia; ^5^ Institute of Evolution University of Haifa Mount Carmel Haifa 34988384 Israel

**Keywords:** adaptive complexes, DNA methylation, environmental stress, *Hordeum spontaneum*, metabolome, plant evolution, transcriptome

## Abstract

At the microsite “Evolution Slope”, Tabigha, Israel, wild barley (*Hordeum spontaneum*) populations adapted to dry Terra Rossa soil, and its derivative abutting wild barley population adapted to moist and fungi‐rich Basalt soil. However, the mechanisms underlying the edaphic adaptation remain elusive. Accordingly, whole genome bisulfite sequencing, RNA‐sequencing, and metabolome analysis are performed on ten wild barley accessions inhabiting Terra Rossa and Basalt soil. A total of 121 433 differentially methylated regions (DMRs) and 10 478 DMR‐genes are identified between the two wild barley populations. DMR‐genes in CG context (CG‐DMR‐genes) are enriched in the pathways related with the fundamental processes, and DMR‐genes in CHH context (CHH‐DMR‐genes) are mainly associated with defense response. Transcriptome and metabolome analysis reveal that the primary and secondary metabolisms are more active in Terra Rossa and Basalt wild barley populations, respectively. Multi‐omics analysis indicate that sugar metabolism facilitates the adaptation of wild barley to dry Terra Rossa soil, whereas the enhancement of phenylpropanoid/phenolamide biosynthesis is beneficial for wild barley to inhabit moist and fungi pathogen‐rich Basalt soil. The current results make a deep insight into edaphic adaptation of wild barley and provide elite genetic and epigenetic resources for developing barley with high abiotic stress tolerance.

## Graphical abstract

Two wild barley populations adapt to hot‐dry Terra Rossa soil, and abutting moist fungi‐rich Basalt soil at the microsite “Evolution Slope”, Tabigha, Israel. Sugar metabolism facilitates edaphic adaptation of wild barley to dry Terra Rossa soil, and phenylpropanoid/phenolamide biosynthesis enables the edaphic adaptation of wild barley to moist and fungi‐rich Basalt soil at epigenomic, genomic, transcriptomic, and metabolomic levels.

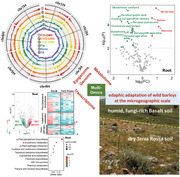

## 1 Introduction

Soil diversity is one the key driving forces of plant evolution for the maintenance of species and ecosystem diversity. Soil also sets the foundation for domestication of the founder crops and their production toward the rise of agriculture and formation of early human civilizations.^[^ ^1^, ^2^ ^]^ Moreover, soil is the living substrate of plants, providing essential physical support, water, mineral elements, and microbiome for their growth and development. The physical, chemical, and biological properties of soil have great influence on the plant growth and development. For instance, poor water retention capacity of sandy soil and frequent drought events tend to drive the evolution and development of deep root plants towards better water use efficiency.^[^ ^3^, ^4^ ^]^ On the contrary, reduced soil aeration and high moisture in siliceous soil facilitates the spread of pathogenic bacteria and fungi, resulting in root infection and deleterious plant health.^[^ ^5^, ^6^ ^]^ Therefore, sustainable and efficient utilization of soil resources could be realized by developing the better crop cultivars adapted to the various types of soils.

Barley has been an important food crop for thousands of years and is now also used as raw material for beverage and feed production. During the domestication of barley, especially after modern breeding and intensive cultivation, the genetic diversity of cultivated barley has been significantly reduced, thus posing the bottleneck of barley breeding for improving biotic and abiotic stress tolerance.^[^ ^7^, ^8^, ^9^, ^10^ ^]^ Wild barley (*Hordeum spontaneum*) has adapted to a wide range of environments differing in soil type, water availability, temperature, and altitude, making it as the ideal genetic resource for the improvement of cultivated barley.^[^ ^11^ ^]^

In the ecological microsite of Evolution Slope (Tabigha, in Upper Galilee, north of the Lake of Galilee; Figure S1, Supporting Information),^[^ ^12^ ^]^ two contrasting types of rocks and soils display dramatically chemical and physical differences: The hard Middle Eocene calcareous limestone has weathered into brown hot and dry Terra Rossa, whereas the abutting Pliocene basalt, is siliceous, moist, and rich in fungi pathogens.^[^ ^13^ ^]^ Previous studies revealed that the wild barley population inhabiting the Terra Rossa soil are much more tolerant to drought stress than those growing in the Basalt soil.^[^ ^14^, ^15^ ^]^ Recently, Bian et al.^[^ ^3^ ^]^ reported that the wild barley Chalk population displays better water deficit tolerance than Basalt population when grown in the dry Chalk soil. In another microclimatic ecosystem—the Evolution Canyon at Mount Carmel of Israel, the population of wild emmer wheat from the wet and shady European Slope exhibited high resistance to pathogens.^[^ ^5^ ^]^ It has therefore been suggested that the wild barley at the Evolution Slope (Tabigha) has evolved adaptive complexes to environmental stresses via long‐term natural selection especially the unique physical, chemical and microbial properties of the soils. However, the underlying mechanisms on how wild barley acquires the stress tolerance remain elusive.

The responses of plants to environmental stresses are complex processes, consisting of the reception of stress signal, the response to stresses, the recovery from stress‐induced injury, the adaptation to stresses, and the transgenerational epigenetic memory of environmental stresses.^[^ ^16^, ^17^ ^]^ Thus, plants need to coordinate these complex processes at multi‐omics levels such as chromatin remodeling, transcriptional regulation, posttranscriptional modulation, posttranslational regulation, protein–protein interaction, metabolic reprograming, and signal transduction. Our understanding of plant response to environmental stresses has been significantly improved due to the rapid development of multi‐omics technologies, such as genome sequencing,^[^ ^18^ ^]^ whole genome bisulfite sequencing (WGBS, DNA methylome),^[^ ^19^ ^]^ transcriptome,^[^ ^20^ ^]^ proteome,^[^ ^21^ ^]^ and metabolome.^[^ ^22^, ^23^ ^]^ Many of these technologies have been extensively employed for prescreening of elite germplasm and identification of novel alleles, epialleles, proteins, and metabolites to facilitate the process of molecular breeding for climate resilient crops.^[^ ^24^, ^25^ ^]^

Strong regulation of DNA methylation on transcriptome was extensively reported when plants are exposed to environmental stresses.^[^ ^26^, ^27^, ^28^ ^]^ Under normal conditions, the transposable elements (TEs) were highly methylated to maintain the genome stability.^[^ ^29^ ^]^ However, TEs can be activated by environmental stresses, a phenomenon that widely occurs from bacteria to plants and animals.^[^ ^30^, ^31^, ^32^ ^]^ Some epigenetic changes are stable and heritable over several generations such as the UV‐C treated *Arabidopsis thaliana* plants.^[^ ^16^ ^]^ In rice (*Oryza sativa*), genome‐wide DNA methylation alterations were observed under drought stress, and 70% of these methylation sites were reversed to original status after water recovery.^[^ ^33^ ^]^ In apple (*Malus domestica*), drought stress was associated with methylation alteration of TEs in a number of genes, including transcription factors (TFs).^[^ ^19^ ^]^ These studies suggested that DNA methylation and TE mobilization can affect the adaptive stress response and genome stability in plants. Therefore, deciphering the interaction of DNA methylome with transcriptome and metabolome in wild relatives of cereal crops such as *Hordeum spontaneum* will benefit the selection and breeding for new cultivars tolerant to abiotic and biotic stresses.

Previously, we performed whole genome resequencing on 13 wild barley accessions from the Evolution Slope (Tabigha). We found strong links between drought tolerance in the Terra Rossa wild barley accessions and the candidate genes associated with drought hormone abscisic acid (ABA) signaling, reactive oxygen species (ROS) metabolism, anti‐oxidative system, and root morphology.^[^ ^18^ ^]^ Here, we conducted a comprehensive multi‐omics analysis on the Terra Rossa and Basalt wild barley populations. The main objectives of this study are to understand how wild barley coordinates multi‐omics adaptive complexes to environmental stresses, to reveal the mechanisms underlying the adaptive strategies of wild barley to the two soil types, and to identify the epialleles, alleles, and metabolites responsible for the edaphic adaptation of wild barley.

## 2 Results

### 2.1 WGBS of Wild Barley Inhabiting the Terra Rossa and Basalt soils

To determine DNA methylation adaptive soil population divergence, we performed WGBS on five representative accessions from each of Terra Rossa and Basalt wild barley populations.^[^ ^18^ ^]^ A total of 1620 Gb clean data were obtained from the ten wild barley accessions, with an average of 162 Gb for each accession (Table S1, Supporting Information). In the DNA methylation analysis, a total of 2085 million uniquely mapped read pairs, which covered 87.1% of the ZQ320 reference genome with an average depth of 17×, were retained after removing the duplicated reads (Table S1, Supporting Information). In plants, DNA methylation occurs in three contexts: CG, CHG, and CHH (H = C, A, or T). After removing cytosine sites with sequencing depths <4 and performing binomial tests using the unmethylated barley chloroplast genome as control, we identified a total of 131408470 methylated CGs (mCG) (94.7% of all CGs), 97714324 mCHGs (77.6% of all CHGs), and 20576899 mCHHs (2.9% of all CHHs) (Table S2, Supporting Information).

### 2.2 Differentially Methylated Regions (DMRs) of Wild Barley from the Terra Rossa and Basalt Abutting Soil Types

**Figure 1:**
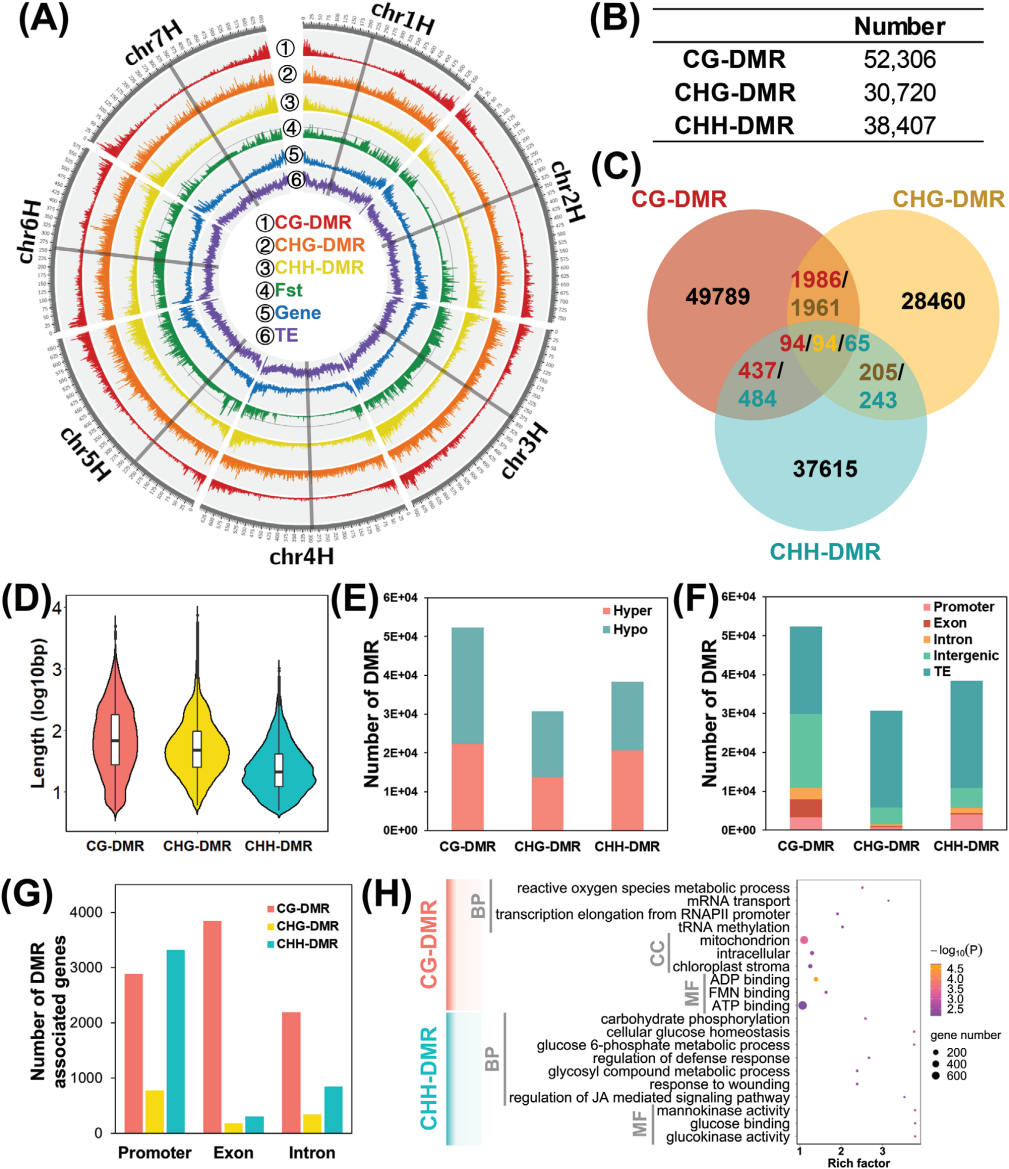
Differential methylation region (DMR) between Terra Rossa and Basalt soil wild barley populations. A) Density of CG‐DMR, CHG‐DMR, CHH‐DMR, *Fst*, gene and transposon element (TE) across genome. The grey line across each chromosome indicates the centromeric regions. B) The number of detected DMR. C) Venn plot of three types DMR. D) The violin plot of length of three types DMR. E) The number of hyper‐ and hypo‐DMR. F) The distribution of DMR in different genomic regions. G) The number of genes associated with DMR in promoter, exon, and intron. H) Gene ontology (GO) enrichment of DMR‐associated genes.

We then investigated DMRs between the two wild barley populations to examine the DNA methylation variation in edaphic adaptation to the two soil types (Terra Rossa vs Basalt). In total, 121433 DMRs were identified, including 52306 CG‐DMRs, 30720 CHG‐DMRs, and 38407 CHH‐DMRs (**Figure** **1**A–C). The average length of DMRs is around 66, 48, and 21 bp in context of CG, CHG, and CHH, respectively. The most different types of DMRs did not overlap with each other (Figure 1C), which may be explained by their short length (Figure 1D). The frequencies of hyper‐ and hypo‐methylation were similar in two wild barley populations, indicating the absence of methylation/demethylation in genome‐wide level (Figure 1E). Moreover, we investigated the distribution of DMRs at the chromosome level by calculating the number of DMRs in 1‐Mb slide windows across the seven chromosomes (Figure 1A). CHG‐DMRs were evenly distributed over each chromosome whereas CG‐DMRs and CHH‐DMRs were highly accumulated in the distal regions of chromosomes (Figure 1A). It is worth noting that the chromosomal distribution pattern of CG‐DMRs and CHH‐DMRs was analogous to that of the genes (Figure 1A). The number of CG‐DMRs (*r* = 0.668, *p* < 0.001) and CHH‐DMRs (*r* = 0.664, *p* < 0.001) was positively correlated with the gene number (Figure S2, Supporting Information). Notably, we also examined the distributions of three DMR types in different genomic regions—promotor, exon, intron, intergenic, and TE. A large number of CG‐DMRs (10908) and CHH‐DMRs (5737) were detected in the gene body and promoter regions, whereas a much lower number of CHG‐DMRs (1497) were located at those regions (Figure 1F). These results suggest potentially important roles of CG‐DMRs and CHH‐DMRs in adaptive functional diversification and regulation of genic regions between the two soil populations of wild barley.

In total, we identified 10478 DMR‐genes, including 7558 CG‐DMR‐genes, 1253 CHG‐DMR‐genes, and 4237 CHH‐DMR‐genes (Figure 1G; Figure S3A,B, Supporting Information). Most of DMRs in the promoter region were in CG (2888) and CHH (3324) context, while CG‐DMRs (5467) were the major type in the gene body region (Figure 1G). Then, we examined the distribution of DMRs in promoter and gene body. In the promoter region (3 kb upstream of the gene body), the CG‐DMRs and CHG‐DMRs were distributed evenly, whereas the CHH‐DMRs were mainly found close to the transcriptional start site (TSS) (Figure S3C, Supporting Information). In the genebody, the CG‐DMRs and CHH‐DMRs were mainly located at the last exon (Figure S3D, Supporting Information). Gene Ontology (GO) analysis was performed to visualize the enrichment of GO term within CG‐DMR‐genes and CHH‐DMR‐genes. Interestingly, CG‐DMRs were mainly enriched in the genes related with the fundamental processes, such as ATP production, transcription, and translation, whereas CHH‐DMR‐genes were enriched in metabolic processes, including carbon and sugar metabolism and stress response pathway (Figure 1H).

### 2.3 The Association between Genome and DNA Methylation

Phylogenetic analysis of the ten wild barley accessions was conducted using single nucleotide polymorphism (SNPs) generated by genome resequencing and RNA‐sequencing, respectively. Additionally, principal component analysis (PCA) and population structure analysis were also performed based on the genome resequencing data. All these results indicated that the wild barley accessions (Basalt 59/60/63 vs Terra Rossa 161/166/169) from the distal regions of the two soil types were completely separated from each other, while the accessions from the boundary regions (Basalt 99 and Terra Rossa 109/110) were found to be intermediate types (Figure S4, Supporting Information). Genomic divergence between the two wild barley populations was estimated by calculation of fixation index *Fst*. High *Fst* values were frequently observed across the barley Chromosomes 3H, 5H, and 6H, indicating the nucleotide difference in these genomic regions between the two wild barley populations (Figure 1A). Interestingly, we found more DMRs in these regions compared with their adjacent regions, that is, 450–500 Mb in Chromosome 3H, 75–100 Mb in Chromosome 5H, and 350–400 Mb in Chromosome 6H. To investigate the relationship between the DMRs numbers and *Fst* values, the correlation analysis was performed between these two parameters. Consistently, there was a significantly positive correlation between *Fst* and the number of CG‐DMRs (*r* = 0.392, *p* < 0.001), CHG‐DMRs (*r* = 0.426, *p* < 0.001), and CHH‐DMRs (*r* = 0.237, *p* < 0.001) (Figure S5A–C, Supporting Information). Moreover, we identified 10478 DMR‐genes and 2095 DSR‐genes, among which 984 genes were shared by the two wild barley populations (Figure S5D, Supporting Information). These results suggest that DMRs may be associated with genomic variation in these wild barley populations.

We also found that the Terra Rossa population had much higher positive Tajima'D value in Chromosomes 3H, 5H, 6H, and 7H, whereas the Basalt population showed lower negative Tajima'D value in Chromosomes 1H, 3H, 6H, and 7H (Figure S6, Supporting Information). These results indicated that Terra Rossa population is under balancing selection, but Basalt population is under directional selection. Consistently, the genetic diversity of Basalt population was lower than that of Terre Rossa population (Figure S7, Supporting Information).^[^ ^18^ ^]^

### 2.4 Transcriptome and Metabolome Analysis of the Two Wild Barley Soil Populations

**Figure 2:**
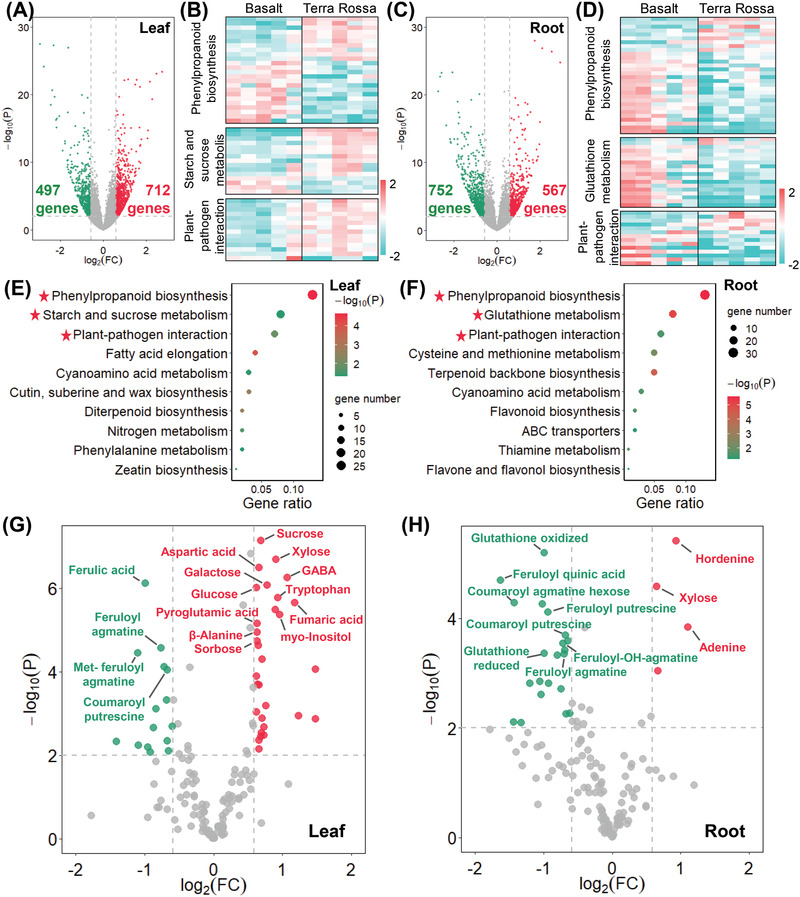
Transcriptome and metabolome analysis of barley accessions from Basalt and Terra Rossa soils. A,C) Volcano plot of differential expressed genes (DEGs) in leaf and root. *X* axis represents the fold change of gene expression (Terra Rossa accessions/Basalt accessions). *Y* axis represents –log_10_ transformed *p*‐value. Fold change >1.5‐fold and *p* < 0.01 means the presence of significant difference in gene expression levels between two populations. The red and green dots represent higher expression in Terra Rossa and Basalt accessions, respectively. B,D) Heatmap of DEGs. E,F) KEGG enrichment of DEGs in leaf and root. G,H) Volcanic plot of fold change in metabolite levels. *X* axis represents the fold change of metabolite levels (Terra Rossa / Basalt). *Y* axis represents –log_10_ transformed *p*‐value. Fold change >1.5‐fold and *p* < 0.01 indicate significant difference of metabolites between two populations. The red and green dots represent high levels of metabolites in the Terra Rossa and Basalt accessions, respectively.

To compare the difference in transcriptional profiles between the two wild barley soil populations, we performed RNA‐sequencing in leaf and root of ten accessions, five in each soil population, resulting in a total of 3.11 billion clean reads (431.97 Gb). On average, the mean clean reads of 51758082 were obtained for each accession, and 87.48% of the clean reads were mapped to the ZQ320 reference genome.^[^ ^34^ ^]^ Using quantitative real‐time PCR (qRT‐PCR), the expression levels of 15 randomly selected differentially expressed genes (DEGs) were found to be similar to those obtained in RNA‐seq analysis results, indicating the reliability and reproducibility of the transcriptome dataset (Figure S8, Supporting Information). Volcano plots of leaf and root of the Terra Rossa accessions showed the upregulation of 712 and 567 DEGs, and downregulation of 497 and 752 DEGs in comparison with the Basalt accessions, respectively (**Figure** **2**A,C). Kyoto Encyclopedia of Genes and Genomes (KEGG) analysis showed that DEGs were enriched in “phenylpropanoid biosynthesis” and “plant‐pathogen interaction” in both leaf and root (Figure 2E,F). In “phenylpropanoid biosynthesis”, the expression levels of 24 genes encoding peroxidase were higher in the Basalt accessions than those in the Terra Rossa accessions (Figure 2B,D; Table S3, Supporting Information). In “plant‐pathogen interaction”, three genes encoding pathogenesis‐related protein 1 displayed significantly higher expression levels in roots of the Basalt accessions than in those of the Terra Rossa accessions (Figure 2D; Table S3, Supporting Information). The gene encoding cyclic nucleotide‐gated ion channel 4 (CNGC4) exhibited higher expression level in roots of the Basalt accessions (Figure 2D; Table S3, Supporting Information). Another KEGG enrichment of DEGs in roots was “Glutathione metabolism”, in which 14 out of the 16 genes encoding glutathione S‐transferase (GST) showed higher expression levels in roots of the Basalt accessions (Figure 2D; Table S3, Supporting Information). Taken together, the pathogen resistance associated pathways are more active in wild barley accessions from the moist Basalt soil than those from the dry Terra Rossa soil.

By contrast, many DEGs in “starch and sucrose metabolism,” including two genes encoding trehalose‐phosphate synthase (TPS) and trehalose‐phosphate phosphatase (TPP), displayed higher expression levels in the leaves of the Terra Rossa accessions (Figure 2B; Table S3, Supporting Information). Additionally, four hydrolase genes, such as beta‐glucosidase, beta‐amylase, and endoglucanase, displayed higher expression in the leaves of the Terra Rossa accessions (Figure 2B; Table S3, Supporting Information). These hydrolases catalyze the hydrolysis of starch or cellulose to produce low molecule sugars such as glucose.^[^ ^35^, ^36^ ^]^ Our results indicate that the Terra Rossa accessions may have higher capacity in sugar production than the Basalt accessions.

The changes in genome, transcriptome, and proteome will eventually alter the metabolome, which is regarded as a mirror to phenotype.^[^ ^37^ ^]^ Therefore, we analyzed the metabolite profiles in the two wild barley soil populations using gas chromatography‐mass spectrometry (GC‐MS) and ultra‐performance liquid chromatography coupled with quadrupole‐time‐of‐flight mass spectrometry (UPLC‐Q‐TOF/MS). In total, 157 and 147 metabolites were identified in leaf and root, respectively (Tables S4 and S5, Supporting Information). Partial least squares discriminant analysis (PLS‐DA) clearly separated the metabolite profiles between the two wild barley populations (Figure S9A,B, Supporting Information). VIP scores plot and volcano plot showed that the concentrations of primary metabolites, such as sugars (sucrose, xylose, galactose, and glucose), organic acids in TCA cycle (citric acid, malic acid, and fumaric acid), and amino acids (glutamic acid, aspartic acid, *γ*‐aminobutyric acid, tryptophan), were higher in leaves of the Terra Rossa accessions. On the other hand, both leaves and roots of the Basalt accessions exhibited higher concentrations of phenolamide and its derivatives, including coumaroyl putrescine and feruloyl agmatine (Figure 2G,H; Figure S9C,D, Supporting Information). In addition, higher concentration of glutathione was observed in the roots of Basalt accessions (Figure 2H).

We also found that the metabolomic differences between the two wild barley populations are well explained by the DEGs. The Terra Rossa accessions exhibited higher sugar concentrations in leaves (Figure 2G), which was in accordance with the higher transcriptional activity of hydrolase genes (Figure 2B; Table S3, Supporting Information). More accumulation of glutathione in roots of the Basalt accessions (Figure 2H) was associated with the higher expression of *GSTs* (Figure 2D; Table S3, Supporting Information). The higher concentrations of phenolamides in the Basalt accessions (Figure 2G,H) were mostly associated with a more active phenylpropanoid pathway (Figure 2B,D; Table S3, Supporting Information). In summary, both transcriptome and metabolome suggested that the Terra Rossa accessions have higher activity in the primary metabolism, while the secondary metabolism is more active in the Basalt accessions.

### 2.5 DNA Methylation Regulated the Expression of mRNA and lncRNA

**Figure 3:**
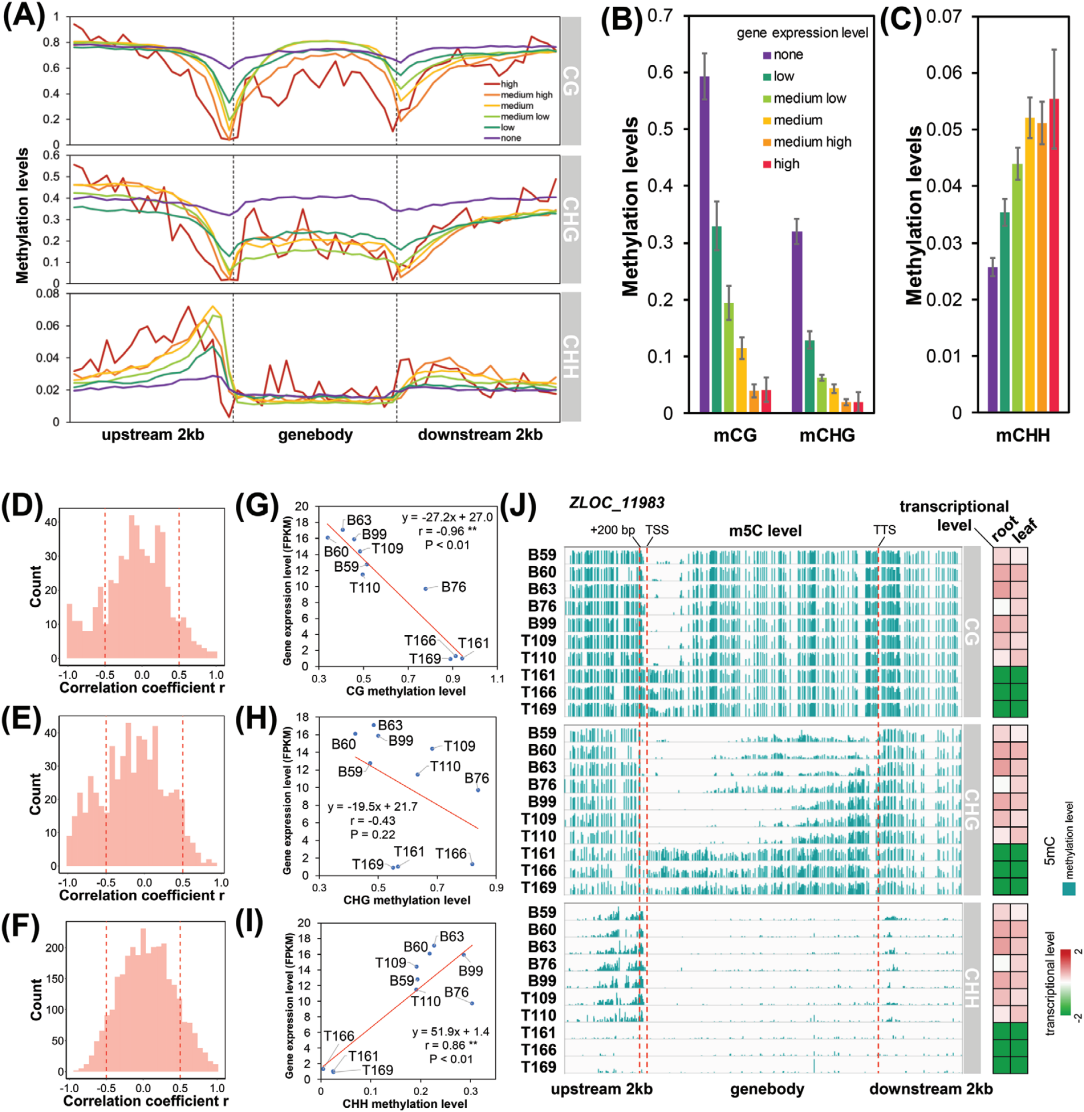
DNA methylation in protein coding genes and its regulation on gene expression. A) Methylation levels within and flanking the genes partitioned by different expression levels. The gene expression was classified into six groups: high, medium high, medium, medium low, low, and none (FPKM value <0.1 was regarded as non‐expressed). B) The average CG and CHG methylation levels in the 100 bp upstream of TSS. C) The average CHH methylation levels from 1000 to 200 bp upstream of TSS. D–F) Distribution of Pearson correlation coefficient between m5C level (D, mCG; E, mCHG; F, mCHH) and transcriptional level. G–I) Correlation analysis between m5C level (G, mCG; H, mCHG; I, mCHH) and transcriptional level of *ZLOC_11983*. J) m5C level and transcriptional level of *ZLOC_11983*.

It is well documented that DNA methylation plays a vital role in the regulation of gene expression.^[^ ^29^ ^]^ The CG and CHG methylation levels decreased sharply in the upstream region close to TSS, with a largest reduction in “high” and “medium high” groups (**Figure** **3**A). The methylation levels in the 100 bp of upstream region were negatively correlated with gene expression levels in the wild barley accessions (Figure 3B). Unlike CG/CHG methylation, CHH methylation level increased gradually from 2000 to 300 bp of upstream region, followed by a dramatic reduction from 300 to 100 bp (Figure 3A). Interestingly, in the upstream region from 1000 to 200 bp, CHH methylation level was positively correlated with gene expression level (Figure 3C). In order to determine the effect of hyper‐ or hypo‐methylation on the expression of individual genes, we performed Pearson correlation analysis between methylation level and gene expression level in 10 wild barley accessions. As a result, the CG methylation level in 0–200 bp upstream of TSS was negatively correlated with the expression level of *ZLOC_11983* (lysine‐specific demethylase 3B) (*r* = −0.96, *p* < 0.01), while CHH methylation level in 200–1000 bp upstream of TSS was positively correlated with the gene expression level (*r* = 0.86, *p* < 0.01) (Figure 3G–J). In addition, number of SNPs in this gene and its flanking region was much less than that of differential methylated cytosine (Figure S10, Supporting Information). It is thus suggested that the differential gene expression may be mostly dependent on differential methylation, rather than genomic variation in those wild barley accessions.

To minimize the false positive results, the differential methylated genes (e.g., CG methylation level <0.2 and >0.8 in at least one accession, respectively) were selected for correlation analysis. Most correlation coefficients showed no statistical significance, indicating that the DNA methylation level was not correlated with gene expression level. However, it is worth noting that the number of the observed negative correlations was more than the expected in CG and CHG context, while in CHH context, the number of the observed positive correlations were more than the expected. For instance, the CG methylation in the promoter region of *ZLOC_7273* (Helicase protein MOM1) and *ZLOC_15741* (cytochrome C oxidase subunit 6b‐1) and the CHG methylation of *ZLOC_11578* (lysine‐specific demethylase JMJ705) suppressed the expressions of these genes, while the CHH methylation promoted the expression of *MLOC_36552* (disease resistance protein RPP13) (Figure S11, Supporting Information).

We also examined the correlation between DNA methylation and lncRNA expression (Figure S12, Supporting Information). Similarly, CG and CHG methylation inhibited the gene expression, while CHH methylation promoted the gene expression. However, the correlations were much stronger in lncRNA than in mRNA (Figure 3D–F; Figure S11G–I, Supporting Information), suggesting a vital role of DNA methylation in the regulation of lncRNA expression. In addition, the PCA of DNA methylation in promoter region of lncRNA showed a clear separation between Basalt59/60/63 and Terra Rossa161/166/169. In contrast, the PCA of DNA methylation in mRNA‐related region did not show separation between these two populations (Figure S13, Supporting Information). Taken together, these results suggest that the regulation of lncRNA expression mediated by DNA methylation may be associated with the adaptation of wild barley to the different types of soils, displaying sharply divergent ecologies.

### 2.6 The Difference in Phenolamide Synthesis between the Two Wild Barley Soil Populations

**Figure 4:**
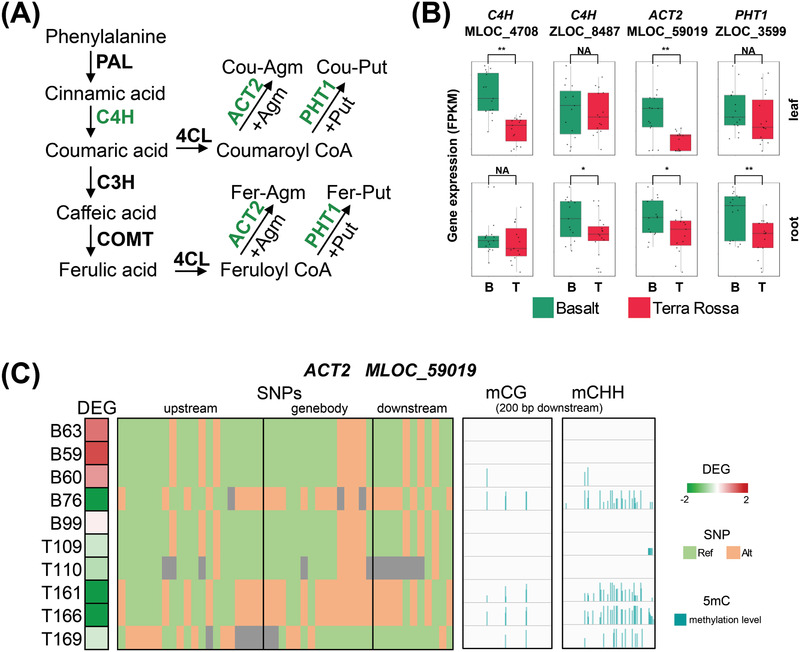
Integration of genomic, DNA methylation, transcriptomic, and metabolomic analysis of phenylamide synthesis pathway. A) The phenylamide synthesis pathway. Green color of the enzymes means their coding genes were differentially expressed among two populations (refer to panel B). B) The gene expression of *C4H*, *ACT2* and *PHT1* in two populations. C) The association among gene expression, local SNP and DNA methylation of *ACT2*. In DEG module, red and green colors represent high and low gene expression, respectively. In SNP module, light green and orange represent reference locus and mutational locus in genebody and its 2 kb upstream/downstream regions aligned with the ZQ320 reference genome. In 5mC module, *y* axis represents the methylation level of cytosine in 200 bp of downstream region.

The phenolamide synthesis pathway (**Figure** **4**A) showed dramatic difference between two wild barley populations at multi‐omics levels (Figure 4A–C). The expression levels of *C4H* (*Cinnamate‐4‐Hydroxylase*), *ACT2* (*Agmatine Coumaroyl Transferase 2*), and *PHT1* (*Putrescine Hydroxycinnamoyl Transferases 1*) were higher in the Basalt accessions (Figure 4B), which was consistent with the relatively higher accumulation of phenolamide (Figure 2G,H). We then took *ACT2* (*MLOC_59019*) and *C4H* (*MLOC_4708*) to examine the relationships among gene expression, local SNP and DNA methylation. The *ACT2* expression in leaf was largely repressed in Basalt76 (FPKM = 0.22) and Terra Rossa161/166 (FPKM = 0.09 and 0.22), while it showed the moderate level in other accessions (FPKM = 3.98–15.77). The repression of gene expression may be associated with the CG and CHH methylation of 200 bp downstream region or the local genomic variation. The *C4H* expression in leaf was much higher in Basalt59/60/63/76 in comparison with all Terra Rossa accessions (Figure S14A, Supporting Information). The *C4H* expression was associated with local SNPs; however, no apparent correlation was detected between gene expression and DNA methylation in either promoter or gene body region (Figure S14B–D, Supporting Information). Interestingly, most of SNPs in the downstream region seem to be associated with *C4H* expression, suggesting that the local genomic variation may contribute to activation or suppression of the related genes.

## 3 Discussion

Climatic, geological, edaphic, and environmental stresses are major driving forces for the evolution of living organisms. The studies on evolutionary microsites in Israel provided excellent examples of the adaptation to micro ecological environment from viruses and bacteria, through fungi, plants, and animals.^[^ ^38^, ^39^, ^40^, ^41^, ^42^, ^43^, ^44^ ^]^ Recently, Wang et al.^[^ ^18^ ^]^ and Bian et al.^[^ ^3^ ^]^ revealed that genomic divergence plays a crucial role in edaphic adaptation of wild barley. Both studies identified novel genes and alleles associated with ABA signaling and root architecture, which facilitates the wild barley to adapt to the dry Terra Rossa and Chalk soils. Epigenetic variations induced by environmental stresses are usually transgenerational inherited,^[^ ^45^ ^]^ which may be useful in crop breeding by improving stress tolerance. However, these are not validated at multi‐omics levels in wild barley.

### 3.1 Epigenomic and Genomic Variations Contribute to the Sharp Divergence between Two Wild Barley Abutting Geological and Edaphic Populations at the Evolution Slope (Tabigha)

Plant growth and stress response are mediated by the complex interactions of genotype and environmental stresses. The understanding of the complex adaptive divergent evolution between the two wild barley populations inhabiting the different soil types has been much improved by the rapid development of multi‐omics technologies.^[^ ^18^, ^19^, ^20^, ^21^, ^22^, ^46^, ^47^ ^]^ The question on how multi‐omics coordinate and interact with each other remains elusive. It has been widely accepted that DNA methylation in the promoter region is one of the epigenetic approaches to regulate (mostly suppress) gene expression.^[^ ^29^ ^]^ Here, we found that the high CG and CHG methylation of the promoter, especially at 0–200 bp upstream of TSS, was associated with low expression of the genes (Figure 3A,B). These differentially methylated genes were enriched in the pathways involved in the fundamental processes, such as ATP production, RNA transcription, and processing (Figure 1H). For example, lysine‐specific demethylases (ZLOC_11983, lysine‐specific demethylase 3B; ZLOC_11578, lysine‐specific demethylase JMJ705) are responsible for histone demethylation, which can regulate the expressions of genes related with flowering time, thus providing prezygotic reproductive isolation and defense response.^[^ ^48^, ^49^ ^]^ JMJ705 can also remove H3K27me3 from defense‐related genes thereby increasing the gene expression during fungal and microbe pathogen infection.^[^ ^49^ ^]^ In three wild barley accessions Basalt59/60/63, demethylation of ZLOC_11578 (JMJ705) promoted the gene expression (Figure S11C, Supporting Information), which may confer wild barley with pathogen resistance for better adaptation to the moist fungi and pathogen‐rich Basalt soil. Cytochrome c oxidase (ZLOC_15741, cytochrome c oxidase subunit 6b‐1) is the last enzyme in the respiratory electron transport chain for ATP production,^[^ ^50^ ^]^ and the hypomethylation induced higher expression of ZLOC_15741 may be associated with higher plant growth and yield of the wild barley in Basalt soil.^[^ ^14^ ^]^

Unlike CG and CHG methylation, CHH methylation was enriched in the regions close to gene body, with a peak of methylation in around 300 bp upstream of TSS (Figure 3A). Similar phenomenon has been reported in maize (*Zea mays*)^[^ ^51^ ^]^ and apple.^[^ ^19^ ^]^ This region was called as CHH island, which may function in adjacent transposon repression to facilitate the initial transcription.^[^ ^51^ ^]^ Here, we found that higher CHH methylation positively regulate the transcription of *MLOC_36552* (encoding disease resistance protein RPP13) in the Basalt wild barley accessions. It was reported that RPP13 is an NBS‐LRR type R protein, and confers resistance to mildew disease in *Arabidopsis*.^[^ ^52^ ^]^ Thus, the increased expression of *MLOC_36552* may improve the disease resistance of the wild barley accessions in Basalt soil. Furthermore, the differential CHH methylations between the two soil populations were highly distributed in the region of CHH island (Figure 1G; Figure S3C, Supporting Information), suggesting their potential roles in edaphic adaptation in wild barley.

The correlation of DNA methylation with lncRNA expression is higher than that with mRNA (Figure 3D–F; Figure S10G–I, Supporting Information), suggesting a vital role of DNA methylation in regulation of lncRNA expression. The variation in DNA methylation of lncRNA located genomic region showed a clear separation between Basalt59/60/63 and Terra Rossa161/166/169 (Figure S12B–D, Supporting Information). Therefore, these differential methylations associated lncRNA may also contribute to the adaptation of wild barley to two types of soils. It is well documented that lncRNA plays an important functional role in plant abiotic and biotic stress.^[^ ^53^, ^54^ ^]^ However, lncRNAs are mostly not conserved among different plant species,^[^ ^55^ ^]^ so that we did not perform further functional prediction of these lncRNA due to little information about their functional characterization in barley.

DNA methylation is only one of the factors (i.e., transcription factor, microRNA, and local genomic variation) affecting gene expression.^[^ ^19^, ^56^ ^]^ Some SNPs in gene body and flanking regions can alter gene expression.^[^ ^57^, ^58^ ^]^ A genome‐wide analysis of expression quantitative trait loci (eQTLs) revealed that approximately 25% and 50% of gene expression are associated with local eQTLs in maize and sweet potato (*Ipomoea batatas*), respectively.^[^ ^59^, ^60^ ^]^ We also found that the gene expression of *C4H* and *ACT2* was associated with local SNP variation (Figure 4C; Figure S14, Supporting Information). Most of these SNPs were associated with each other due to the strong linkage disequilibrium. Therefore, the exact SNPs leading to the transcriptional changes in *C4H* and *ACT2* and other key DEGs need to be studied in future work.

### 3.2 Improved Sugar Metabolism Facilitates Adaptation of Wild Barley to the Dry Terra Rossa Soil

Drought stress is one of the most severe abiotic stresses restricting crop production worldwide. Plants cope with drought stress through multiple strategies, including osmotic regulation by osmoprotectants such as sugars and metabolites.^[^ ^61^, ^62^ ^]^ In this study, the Terra Rossa accessions showed significantly higher concentrations of many sugars (i.e., glucose, galactose, and xylose), accompanied with higher transcriptional level of hydrolase genes in leaves in comparison with the Basalt accessions. These sugars can serve as vital osmoprotectants for the adaptation of wild barley to drought stress. Additionally, we found more accumulation of glutamic acid in the Terra Rossa accessions. Glutamic acid is a precursor for synthesis of proline, which is known to be a major osmoprotectant in plants under many environmental stresses including drought.^[^ ^63^, ^64^ ^]^ High concentration of glutamic acid can provide sufficient substrate for proline synthesis. Moreover, trehalose is synthesized as a stabilizer of cell structure in response to drought stress.^[^ ^65^ ^]^ The observed higher expression of trehalose synthesis genes *TPP* and *TPS* in leaves of the Terra Rossa accessions may enhance the synthesis of trehalose in wild barley. In addition to transcriptional and metabolomic regulation, the DNA methylation on the genes associated with carbohydrate and sugar metabolism also differed significantly between the two wild barley populations. Taken together, it may be suggested that the enhancement of sugar metabolism facilitates edaphic adaptation of wild barley to dry Terra Rossa soil at the Evolution Slope (Tabigha).

### 3.3 Enhanced Phenolamide Biosynthesis Improves Pathogen Resistance in Wild Barley from Moist and Pathogen Fungi‐Rich Basalt Soil

Plants have evolved a complex defense system against pathogens, such as pre‐formed structures (i.e., cell wall, trichome) and metabolites (i.e., lignin, phenolamide).^[^ ^66^, ^67^ ^]^ It is well known that the phenolamide has strong antifungal activity in plants.^[^ ^68^ ^]^ In this study, we found significantly more accumulation of phenolamide and higher expression of key genes (*C4H*, *ACT2*, and *PHT1*) in both leaves and roots of the Basalt accessions in comparison with the Terra Rossa accessions. Lignin not only serves as a physical barrier to fungi, but also has antifungal activity due to its nature as a phenolic compound.^[^ ^69^ ^]^ Peroxidase is an essential enzyme in biosynthesis of lignin. In this study, a number of genes encoding peroxidase showed higher expression in roots of the Basalt accessions, which may contribute to lignin biosynthesis and antioxidant activity. Furthermore, some “plant‐pathogen interaction” associated genes (*PR1*, *CNGC4*, and *GST*) showed relatively higher expressions in roots of the Basalt accessions. Pathogenesis‐related (PR) proteins are produced as a part of the systemically‐acquired adaptive resistance in plants in response to pathogens and have antimicrobial activity.^[^ ^70^ ^]^ CNGC4 is a calcium transporter, and it is essential for pathogen‐associated molecular patterns (PAMPs) triggering immunity signaling pathway.^[^ ^71^ ^]^ GST can detoxify toxic lipid hydroperoxides that accumulate during microbial infections.^[^ ^72^ ^]^ A recent study revealed that a wheat gene *Fhb7* encoding GST conferred broad resistance to Fusarium species by detoxifying trichothecenes via de‐epoxidation.^[^ ^73^ ^]^ Additionally, we found that the DMR‐genes were enriched in GO terms “regulation of defense response” and “regulation of JA mediated signaling pathway”. It is thus suggested that the methylation status of the genes in these pathways differ between the two wild barley populations, although the effect of methylation difference is still unclear. As *C4H* and *ACT2* are key genes controlling the biosynthesis of phenolamide,^[^ ^74^, ^75^ ^]^ the higher expressions of these genes caused higher accumulation of phenolamide in the Basalt accessions than in Terra Rossa accessions (Figures 2G,H and 4B). Our integrated multi‐omics analysis of *C4H* and *ACT2* demonstrated the potential association among DNA methylation, genomic variation, and gene expression, resulting in significant difference of key metabolites (Figure 4C; Figure S14A and Tables S4 and S5, Supporting Information). Overall, our results revealed that the enhancement of phenylpropanoid/phenolamide biosynthesis and immunity signaling pathway enables the edaphic adaptation of wild barley to moist and fungi and bacteria‐rich Basalt soil at the Evolution Slope (Tabigha).

## 4 Conclusions

Our study demonstrated that the edaphic adaptation of wild barley to the contrasting soil may be regulated at epigenomic, transcriptomic, and metabolomic levels. Notably, inheritable epigenomic (DNA methylation) divergence occurs during long‐term edaphic adaptation of wild barley grown in the Evolution Slope. Some of these DNA methylations were closely associated with the transcriptional levels of genes participating in fundamental biological processes (Figures 1 and 3). Moreover, at transcriptional and metabolomic levels, the key genes and metabolites associated with sugar metabolism and phenolamide biosynthesis may enable wild barley to adapt to dry Terra Rossa soil and moist fungi‐rich Basalt soil, respectively (Figures 2 and 4). Therefore, these elite alleles and epialleles and anti‐fungi metabolites can serve as vital genetic resource for improvement of abiotic and biotic tolerance in cultivated barley and other cereal crops for ensuring sustainable grain production and food security.

## 5 Experimental Section

### Plant Materials

Ten wild barley accessions were collected from Evolution Slope (Tabigha), eastern Upper Galilee, and characterized.^[^ ^18^ ^]^ Five accessions (Terra Rossa 109/110/161/166/169) were from dry Terra Rossa soil, and the others (Basalt59/60/63/76/99) were from moist and pathogen‐rich Basalt soil. The seeds were germinated and the seedlings were hydroponically cultured in a growth chamber (14 h light, 20 °C/10 h dark, 14 °C) in Zhejiang University, Hangzhou, China. The 17‐day‐old seedlings at three‐leaf stage were used for the following experiments.

### WGBS Analysis

WGBS analysis, a technology for determination of DNA methylation status of single cytosines, was performed according to Shen et al.^[^ ^46^ ^]^ with some modifications. Total genomic DNA was extracted from leaves and roots of ten wild barley accessions and the DNA fragments were treated twice with bisulfite using the EZ DNA Methylation‐Gold Kit (Zymo Research, Irvine, CA, USA). Then, the DNA was used for WGBS library construction, and the libraries were sequenced by Biomarker Technologies (Qingdao, China) using Illumina HiSeq 2500 (San Diego, CA, USA). Adapters and low‐quality bases in the WGBS reads were trimmed using Trimmomatic software Version 0.36 (http://www.usadellab.org/cms/). To minimize the interference of nucleotide variation to DNA methylation analysis across these wild barley accessions, a pseudo‐genome sequence of each accession was constructed using resequencing data at 20 × genome coverage.^[^ ^18^ ^]^ Pseudo genome of each accession was constructed by mapping resequencing data to the barley reference genome ZQ320.^[^ ^34^ ^]^ The pseudo‐genomes were used as reference genomes in the DNA methylation analysis. The trimmed reads were mapped to the assembled pseudo genomes using Bismark software version 0.14.5 (https://www.bioinformatics.babraham.ac.uk/projects/bismark/) with default parameters. The methylation information for each cytosine site was extracted after removing the duplicate reads. The cysteines with >4× genome coverage were used in methylation states test, and the methylation states were evaluated by the binomial test with false discovery rate with *p*‐value <0.05. Differentially methylated regions (DMRs) between the two wild barley populations were identified using MOABS software (http://code.google.com/p/moabs/). The identification of a DMR should meet the criteria: (i) the genome coverage of tested cysteine was more than 10; (ii) there are at least three methylated cysteines with <300 bp in distance between adjacent cytosine sites; (iii) the difference in average methylation level was more than 0.4, 0.4, and 0.2 for CG‐DMR, CHG‐DMR, and CHH‐DMR, respectively, with *p*‐value <0.05 in Fisher's exact test. The genes with the gene body and its 3 kb flanking region that overlapped with DMR were termed as DMR‐genes. Gene ontology (GO) analysis of DMR‐genes was performed using GOseq R package. The visualization of methylation status in a certain genomic region was conducted using IGV software Version 2.8.9 (http://software.broadinstitute.org/software/igv/). Principal component analysis of DNA methylation was conducted using R packages FactoMineR v2.4 and factoextra v1.0.7 (https://cran.r‐project.org/).

### Genome Resequencing Analysis

We reanalyzed the genome resequencing data of the 10 wild barley accessions (see “Plant Materials” part) obtained from a previous study.^[^ ^18^ ^]^ Phylogenetic tree was performed using phylip v3.697 (https://evolution.genetics.washington.edu/phylip.html). Principal component analysis of SNPs was conducted using PLINK v1.9 (http://zzz.bwh.harvard.edu/plink/). The population structure of 10 wild barley accessions was analyzed using ADMIXTURE v1.3.0 (https://bioinformaticshome.com/tools/descriptions/ADMIXTURE.html). The genetic diversity Pi and Tajima’ D of each wild barley population was calculated using VCFtools (http://vcftools.sourceforge.net).

### RNA‐Sequencing Analysis

Two tissues (leaf and root) and three biological replicates for each wild barley accession (five accessions from each soil) were used for RNA‐sequencing. Total RNA was extracted using the RNeasy Plant Mini Kit (QIAGEN, Germany). The cDNA libraries were constructed using NEBNext Ultra RNA Library Prep Kit for Illumina (NEB, USA). The libraries were sequenced by Biomarker Technologies (Qingdao, China) using Illumina HiSeq 2500 (San Diego, CA, USA) to generate paired‐end reads. After removing the adapter sequences and low quality reads, the clean reads were mapped to reference genome (ZQ320) using HISAT2 (http://ccb.jhu.edu/software/hisat2). The gene expression was evaluated using String Tie (https://ccb.jhu.edu/software/stringtie), and was normalized using the FPKM method (the numbers of reads per kilobase of exon sequence in a gene per million mapped reads). To validate the reliability of transcriptome data, the gene expressions of 15 randomly selected genes were examined by qRT‐PCR analysis. The qRT‐PCR analysis was conducted using SYBR Green fluorescence and PerfectStartTM Green qRCR SuperMix on a Roche LightCycler 480 sequence detection system. *HvActin* was used as the reference gene. Differential expressed genes (DEGs) between the two wild barley populations were identified using DESeq2_edgeR R package with *p*‐value < 0.01 and the fold change of gene expression more than 1.5‐fold. KEGG analysis of DEGs were conducted to identify the pathway enrichment of DEGs. Volcano plot, heatmap, and KEGG enrichment plot of DEGs were made using R software Version 4.0 (https://www.r‐project.org).

### Metabolome Analysis

The same leaf and root samples collected for RNA‐sequencing were used in metabolome analysis. The metabolites of samples were determined using ultra‐performance liquid chromatography quadrupole time of flight mass spectrometry (UPLC‐Q‐TOF/MS) and gas chromatography mass spectrometry (GC‐MS), according to Tsugawa et al.^[^ ^76^ ^]^ and Lisec et al.^[^ ^77^ ^]^ with some modifications. The extract was prepared according to Lisec et al.^[^ ^77^ ^]^ and dried under vacuum drier. The residue was derivatized with 70 µL *N*‐methyl‐*N*‐(trimethylsilyl) trifluoroacetamide (MSTFA) (Sigma, USA). The product of derivation reaction was analyzed by 7890A/5975C GC‐MS equipped with HP‐5 capillary column (Agilent, USA). The setting of parameters was as follows: the flow rate of helium at rate of 1 mL min^−1^; the temperature was maintained at 80 °C for 2 min, followed by 5 °C per min ramp to 300 °C, and was maintained at 300 °C for 10 min. Mass spectra were acquired with *m*/*z* from 70 to 600 Da. The raw data was processed with AMDIS software (http://chemdata.nist.gov/).

The supernatant (before adding chloroform) of sample extract in GC‐MS analysis was filtered using 0.45 µm membrane filter, and collected for UPLC‐Q‐TOF/MS analysis. UPLC‐Q‐TOF/MS analysis was performed using a UPLC (Waters, USA) equipped with ACQUITY UPLC BEH‐C18 column (Waters, USA). The mobile phases were 0.1% formic acid‐water (A) and 0.1% formic acid‐acetonitrile (B). The linear gradient settings of elution procedure were as follows: 0 min, 1% B; 0.5 min, 1% B; 0.5 min, 25% B; 6.5 min, 35% B; 10 min, 50% B; 13 min, 95% B, 13.5 min, 1% B; flow rate was set to 0.5 mL min^−1^. Mass data was acquired in the mass range of *m*/*z* 100–2000 Da in positive ion mode using AB Triple TOF 5600plus System (AB SCIEX, USA). The raw data were processed with MS‐DIAL software.^[^ ^76^ ^]^

The identification of metabolites was performed using AMDIS and MS‐DIAL software. Partial least squares discriminant analysis (PLS‐DA) analysis, VIP plot and volcano plot were performed using MetaboAnalyst software (v4.0; https://www.metaboanalyst.ca). The metabolites with fold change of metabolite concentration more than 1.5‐fold (*p* < 0.01) were regarded as differential metabolites between the two wild barley soil populations.

### Multi‐Omics Analysis

We performed correlation analysis between the number of DMRs and fixation index (*Fst*) values. The *Fst* values were estimated using genome resequencing data in our previous study.^[^ ^18^ ^]^ The genomic regions with top 5% of *Fst* value were termed as differential sequence regions (DSRs), and the genes in these regions were termed as DSR‐genes. We displayed the venn plot of the number of DSR‐genes and DMR‐genes.

We investigated the relationship between DNA methylation and gene expression at genome‐wide level. According to FPKM value, all genes were divided into six groups: high (FPKM >1000), medium high (100–1000), medium (10–100), medium low (1–10), low (0.1–1), and none (<0.1). The percentage of CG, CHG, and CHH methylation level in gene body and its 2 kb flanking regions (divided into 20 fragments in each section) for each group was calculated. Then, we performed Pearson correlation analysis between methylation level and gene expression level in 10 barley accessions for each methylated gene. CG and CHG methylation in 0–200 bp of promoter, and CHH methylation in 200–1000 bp of promoter were used in correlation analysis in order to minimize the false positive results, and the differential methylated genes were selected and used in this analysis, that is, the gene with CG methylation level <0.2 and >0.8 in at least one accession, respectively; CHG methylation level <0.2 and >0.5; CHH methylation level <0.02 and >0.1).

Finally, we performed multi‐omics analysis of the selected genes and pathway. The gene expression, SNP and DNA methylation in gene body, and its flanking region were displayed. The SNPs were acquired using genome resequencing data in our previous study (Wang et al., 2018). The visualization of DNA methylation was displayed using IGV software Version 2.8.9 (http://software.broadinstitute.org/software/igv/).

## Conflict of Interest

The authors declare no conflict of interest.

## Author Contributions

S.C. and Q.S. contributed equally to this work. G.Z. and E.N. designed and supervised this study. S.C., Q.S., and Y.H. performed the experiments. S.C., Z.H., and D.W. performed the data analysis. S.C., Z.H.C., E.N., and G.Z. wrote the manuscript with inputs from other authors.

## Supporting information

Supporting Information

Supporting Information

## Data Availability

The original data files of the WGBS and transcriptome are available on NCBI BioProject PRJNA688011 and PRJNA665933, respectively.
