# Supplementary material for: Multi‐Omics Analysis Reveals the Mechanism Underlying the Edaphic Adaptation in Wild Barley at Evolution Slope (Tabigha)
Source: Adv Sci (Weinh). 2021 Aug 13;8(20):2101374. doi: 10.1002/advs.202101374 (PMC8529432; doi:10.1002/advs.202101374)
Supplement: Supplementary file 1 — Supporting Information [file ADVS-8-2101374-s002.pdf]

© 2021 Wiley-VCH GmbH

ADVANCED SCIENCE
Open Access

Supporting Information

for *Adv. Sci.*, DOI: 10.1002/advs.202101374

Multi-Omics Analysis Reveals the Mechanism Underlying the Edaphic Adaptation in Wild Barley at Evolution Slope (Tabigha)

Shengguan Cai, Qiufang Shen, Yuqing Huang, Zhigang Han, Dezhi Wu, Zhong-Hua Chen, Eviatar Nevo, and Guoping Zhang**

Supporting Information

Multi-omics reveals the mechanism underlying the edaphic adaptation in wild barley at Evolution Slope (Tabigha)

Shengguan Cai†, Qiufang Shen†, Yuqing Huang, Zhigang Han, Dezhi Wu, Zhong-Hua Chen, Eviatar Nevo, Guoping Zhang**

Dr. S. Cai, Dr. Q. Shen, Dr. Y. Huang, Dr. Z. Han, Dr. D. Wu, Prof. G. Zhang
College of Agriculture and Biotechnology
Zhejiang University
Hangzhou 310058, China
Email: zhanggp@zju.edu.cn

Dr. Y. Huang
Institute of Crop Science
Hangzhou Academy of Agricultural Sciences
Hangzhou 310024, China

Prof. Z. Chen
School of Science
Western Sydney University
Penrith, NSW 2751, Australia

Prof. Z. Chen
Hawkesbury Institute for the Environment
Western Sydney University
Penrith, NSW 2751, Australia

Prof. E. Nevo
Institute of Evolution
University of Haifa
Mount Carmel, 34988384 Haifa, Israel
Email: nevo@evo.haifa.ac.il

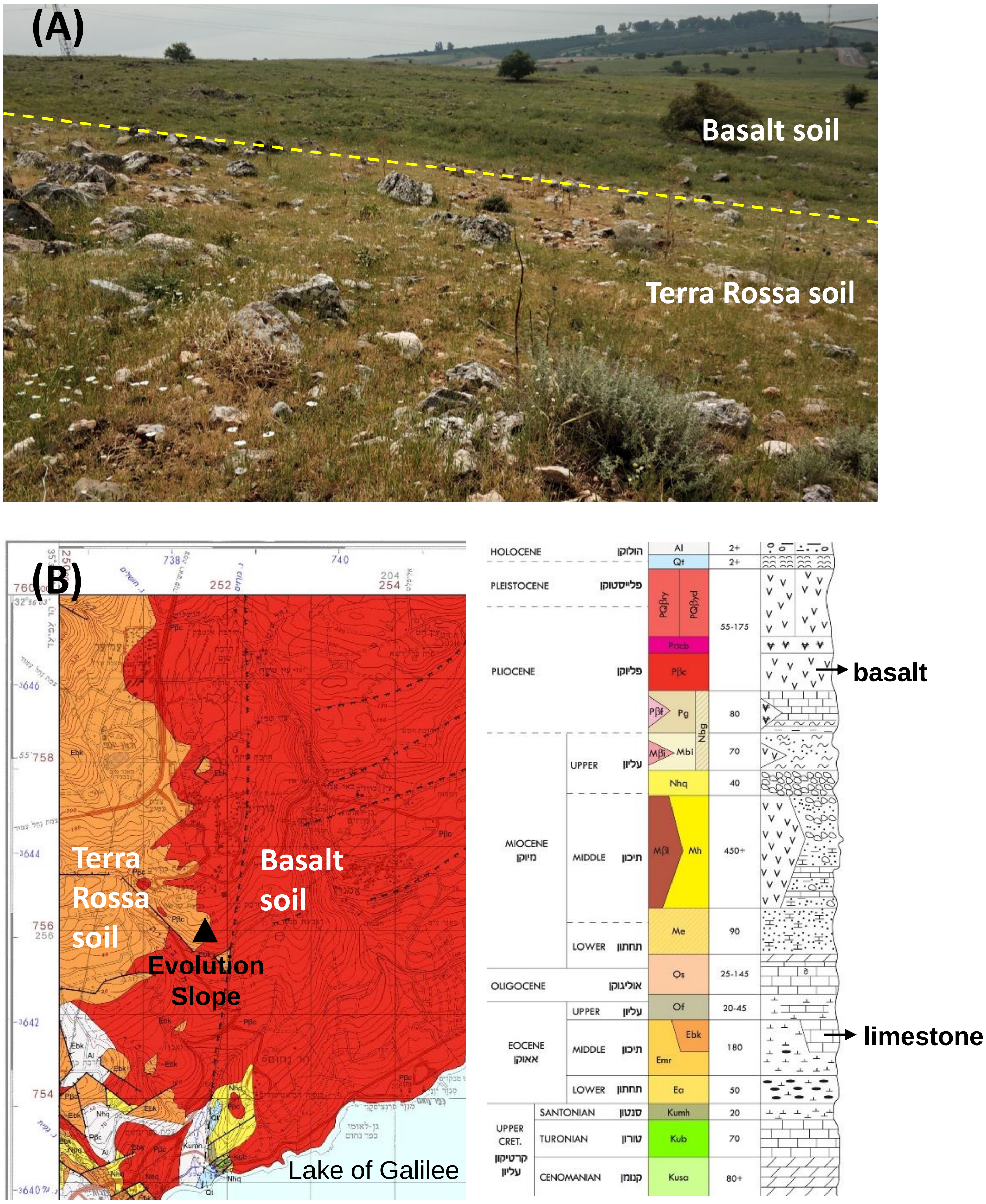

Figure S1. Basalt and Terra Rossa soils in Evolution Slope (Tabigha). A) Terra Rossa soil is dry, hard Middle Eocene limestone, and Basalt soil is humid, fungi-rich, Pleistocene volcanic soil. The yellow line is a geological-edaphic line, separating dramatically divergent wild barley (*Hordeum spontaneum*). Note that the wild barley in the Terra Rossa is yellow which indicates earlier flowering that could provide temporal prezygotic reproductive isolation, i.e. sympatric speciation and a new wild barley species on the Basalt. **B)** Geological map of Teverya in Israel. The map was modified and edited by A. Sneh in Geological Institute of Israel (https://www.gov.il/en/departments/general/map-1-50000). The brown represents Middle Eocene limestone, and the red represents Pliocene volcanic basalt.

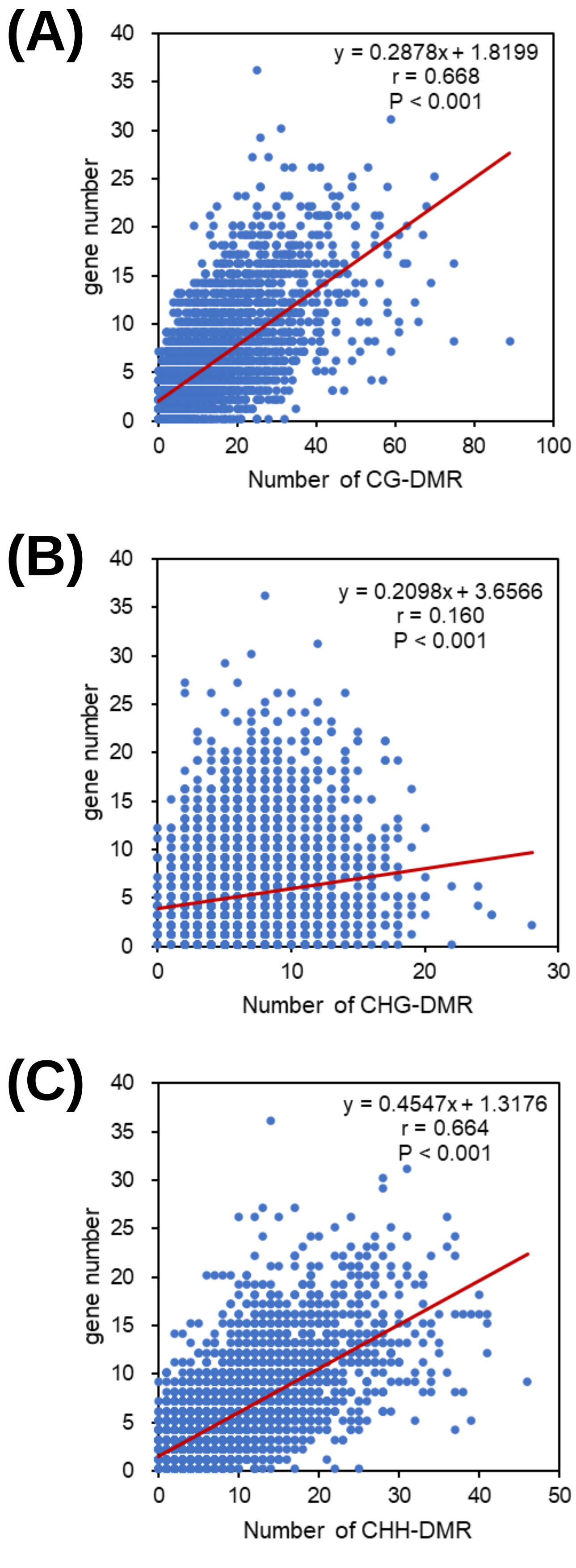

Figure S2. The correlation between gene number and the number of DMRs in three contexts. The DMR number and gene number was counted in a slide window of 1 Mb.

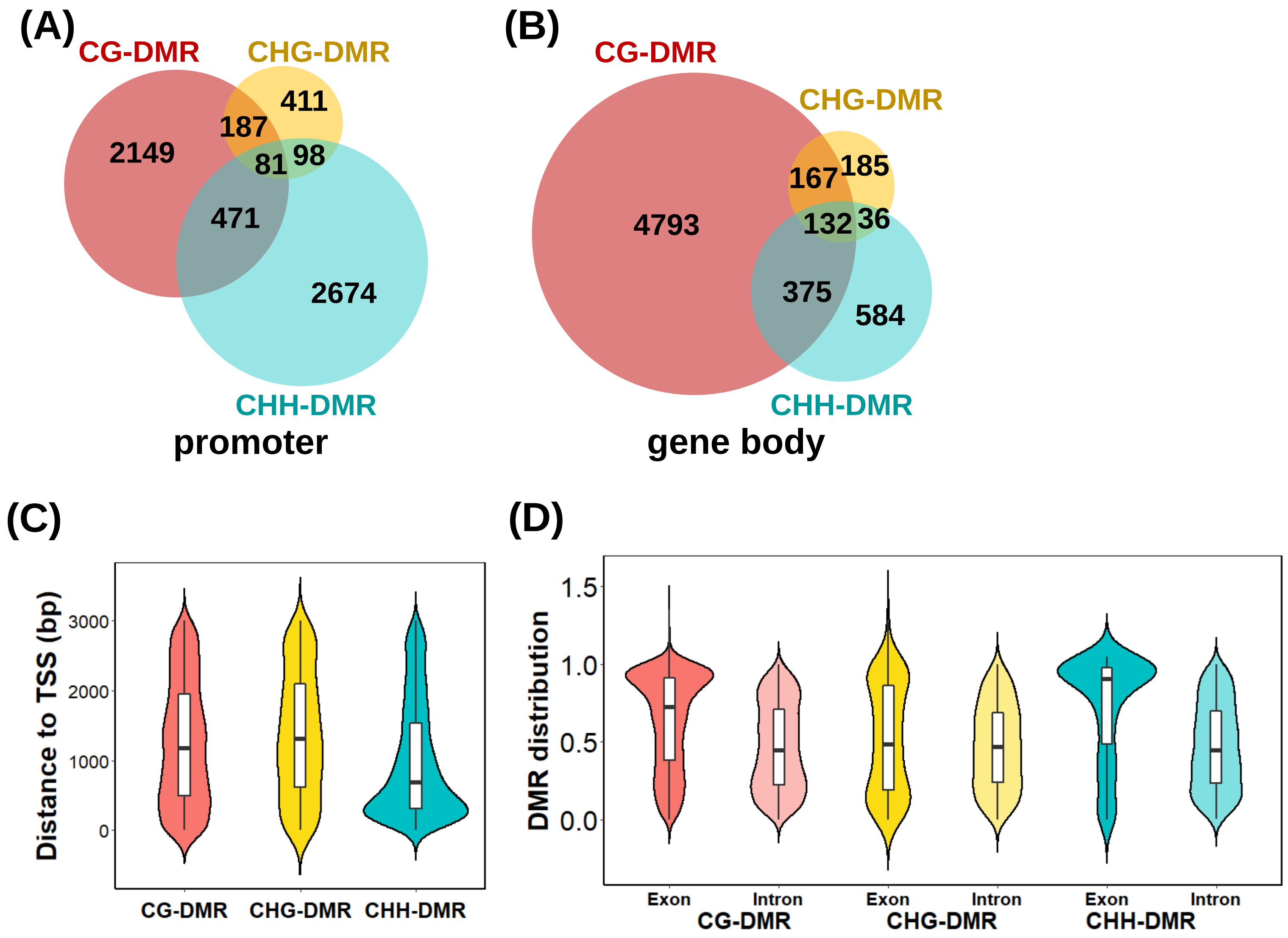

Figure S3. The statistics of DMR-associated genes. A,B) venn plot of three types DMR in promoter (**A**) and gene body (**B**). **C)** violin plot of DMR located at 3 kb upstream of transcriptional start site (TSS). **D)** violin plot of DMR located at exon and intron.

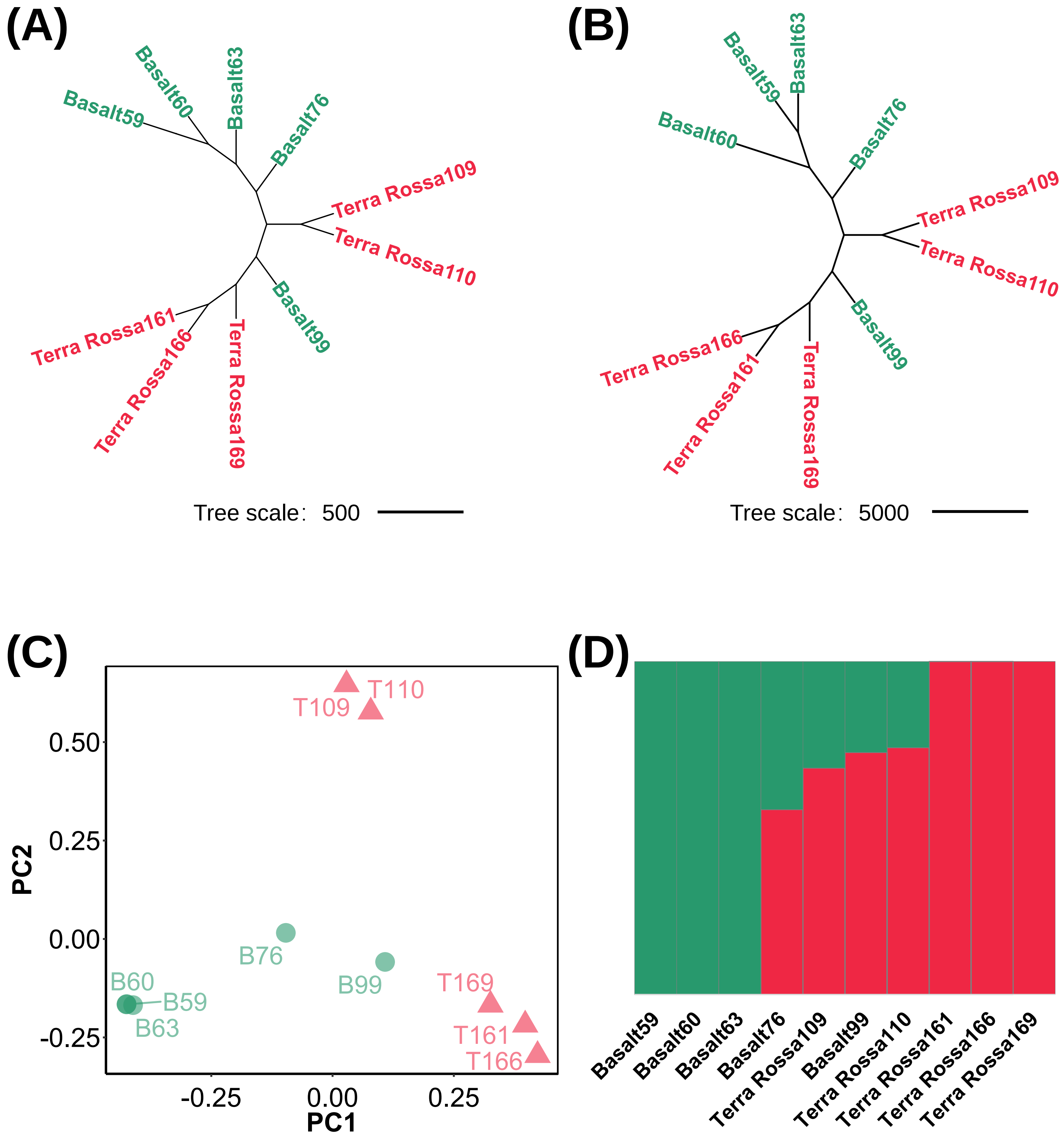

Figure S4. Phylogenetic tree, principal component analysis (PCA) and population structure based on genome and transcriptome. A,B) The tree was constructed based on SNPs from resequencing data (**A**) and transcriptome data (**B**), using neighbor-joining method with 1,000 bootstrap replications. **C)** PCA plot of 10 wild barley accessions. **D)** population structure of two wild barley soil populations.

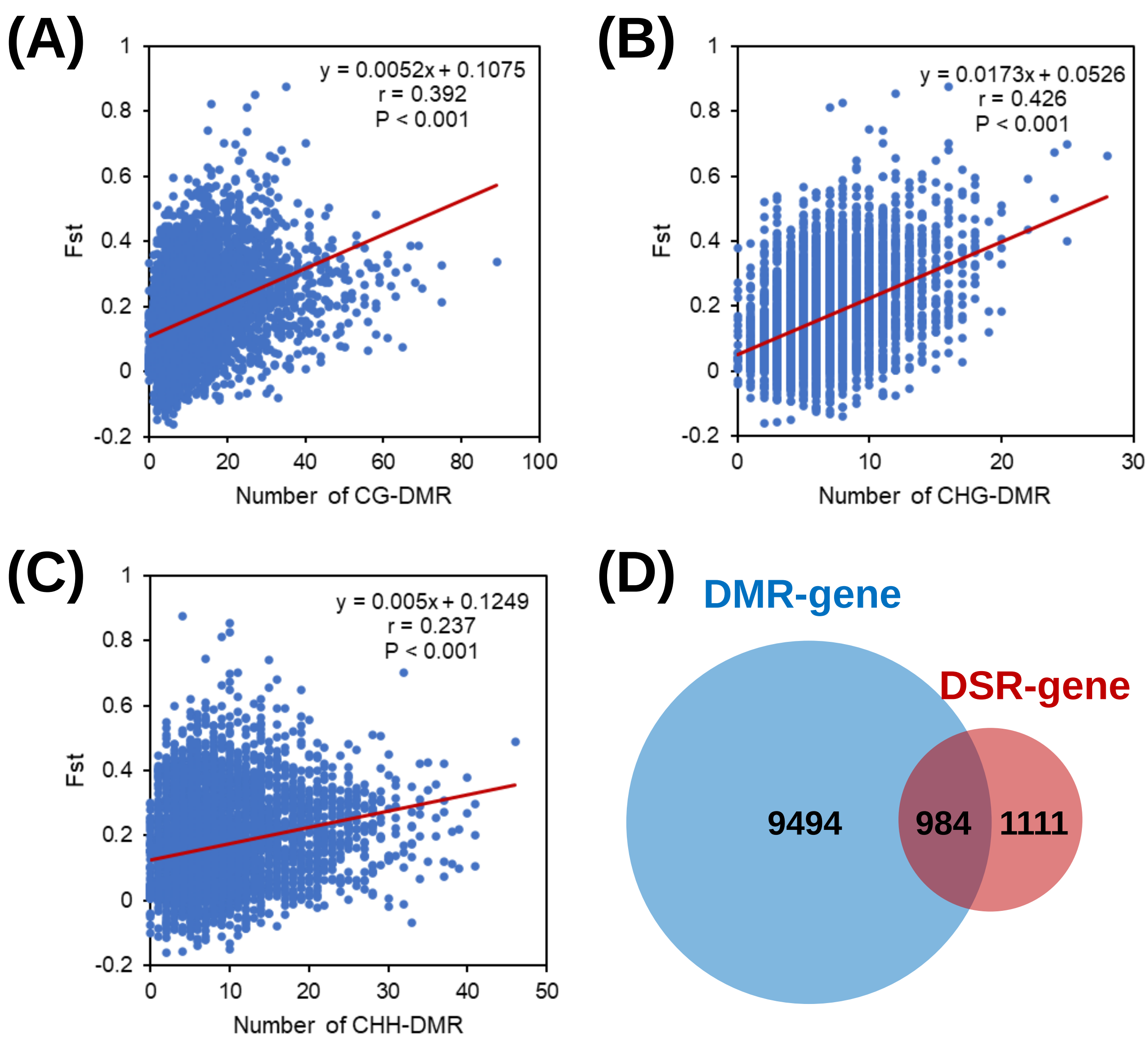

Figure S5. The association between DMRs and DSRs. A-C) The correlations between Fst and the number DMRs in three contexts. The DMR number and Fst was calculated in a slide window of 1 Mb. **D)** Venn plot of DMR-associated genes (DMR-gene) and DSR-associated genes (DSR-gene).

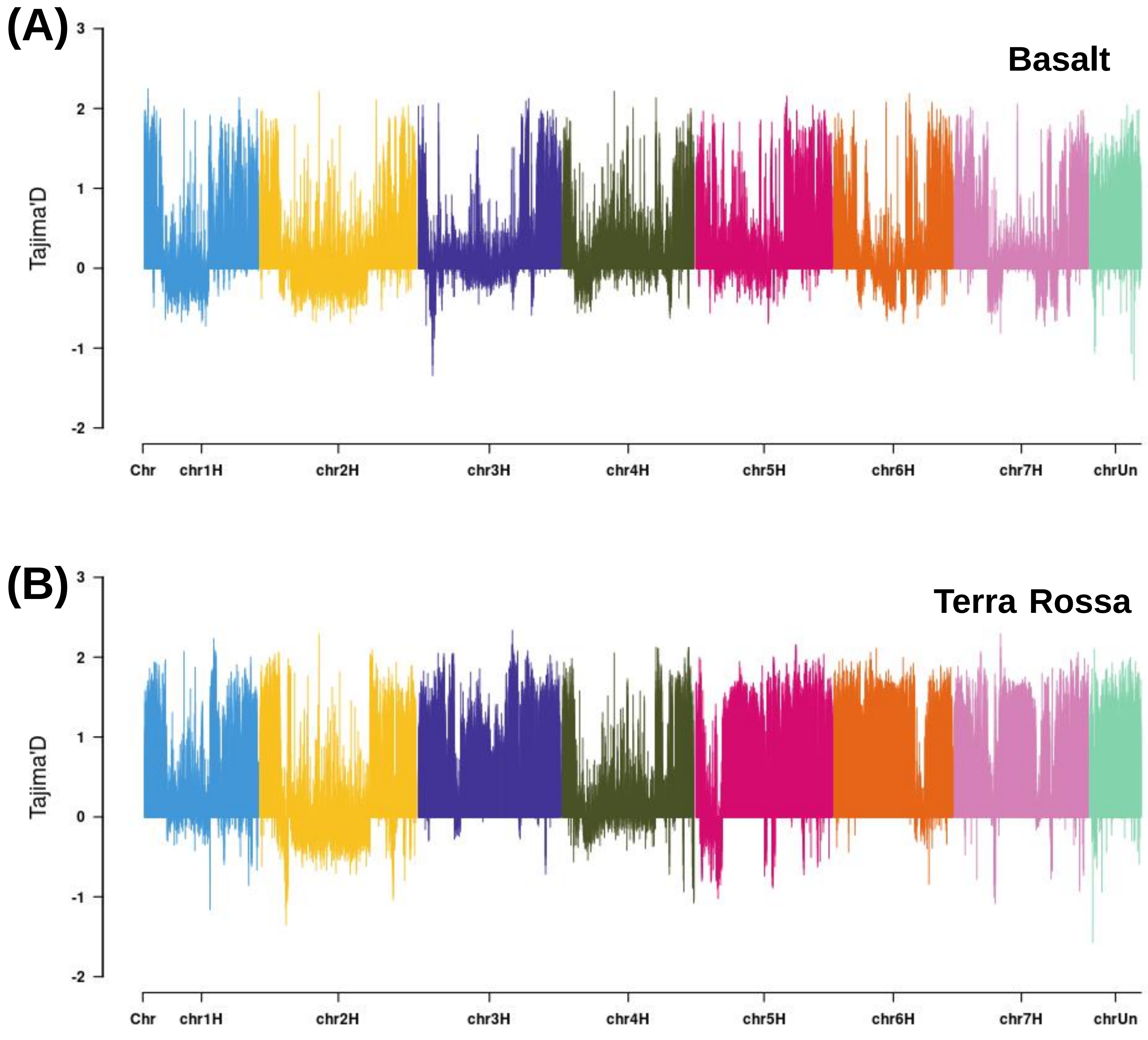

Figure S6. Tajima’D in Basalt and Terra Rossa wild barley soil populations. A) Basalt. **B)** Terra Rossa.

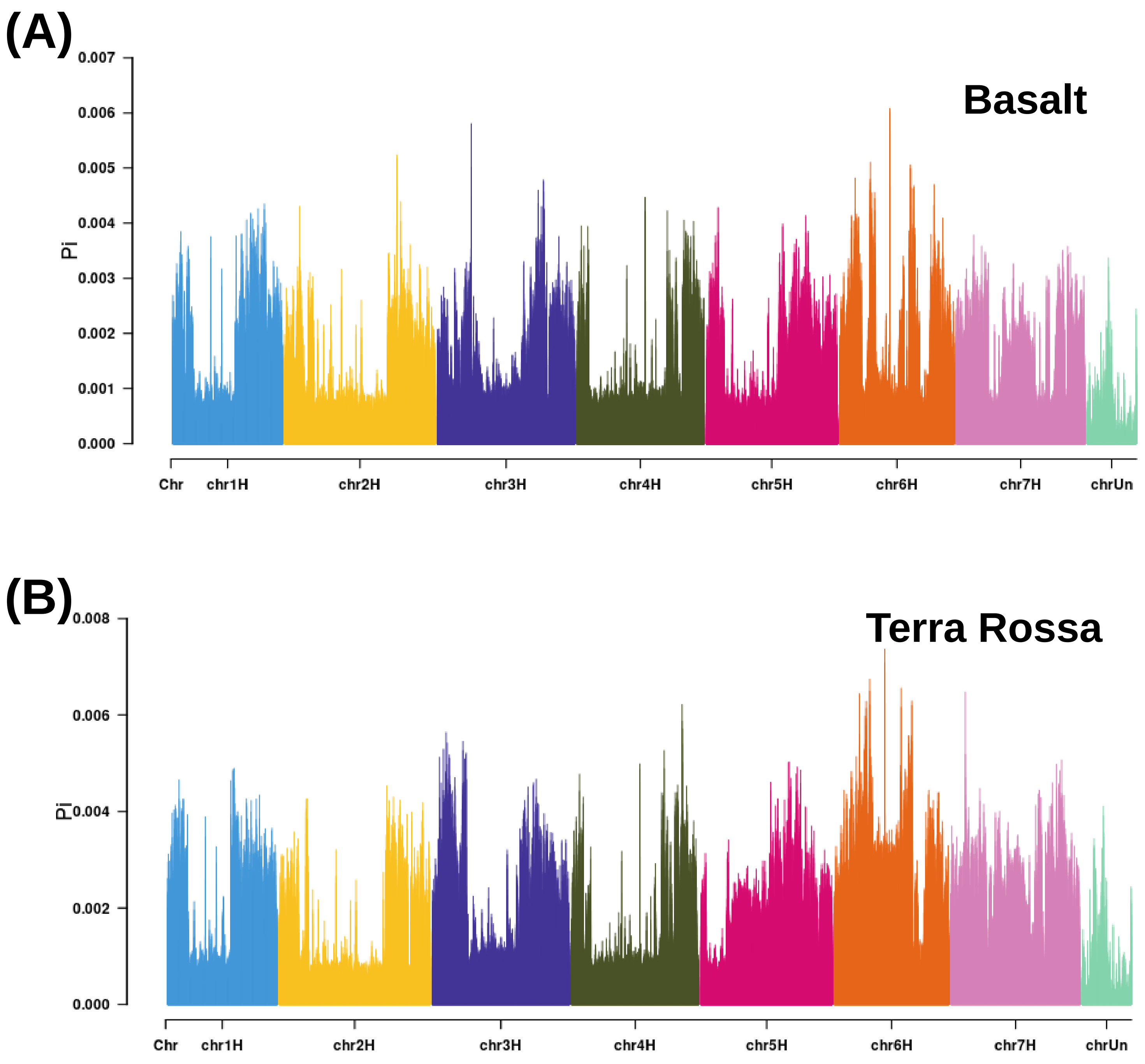

Figure S7. Genetic diversity (π) in Basalt and Terra Rossa wild barley soil populations. A) Basalt. **B)** Terra Rossa.

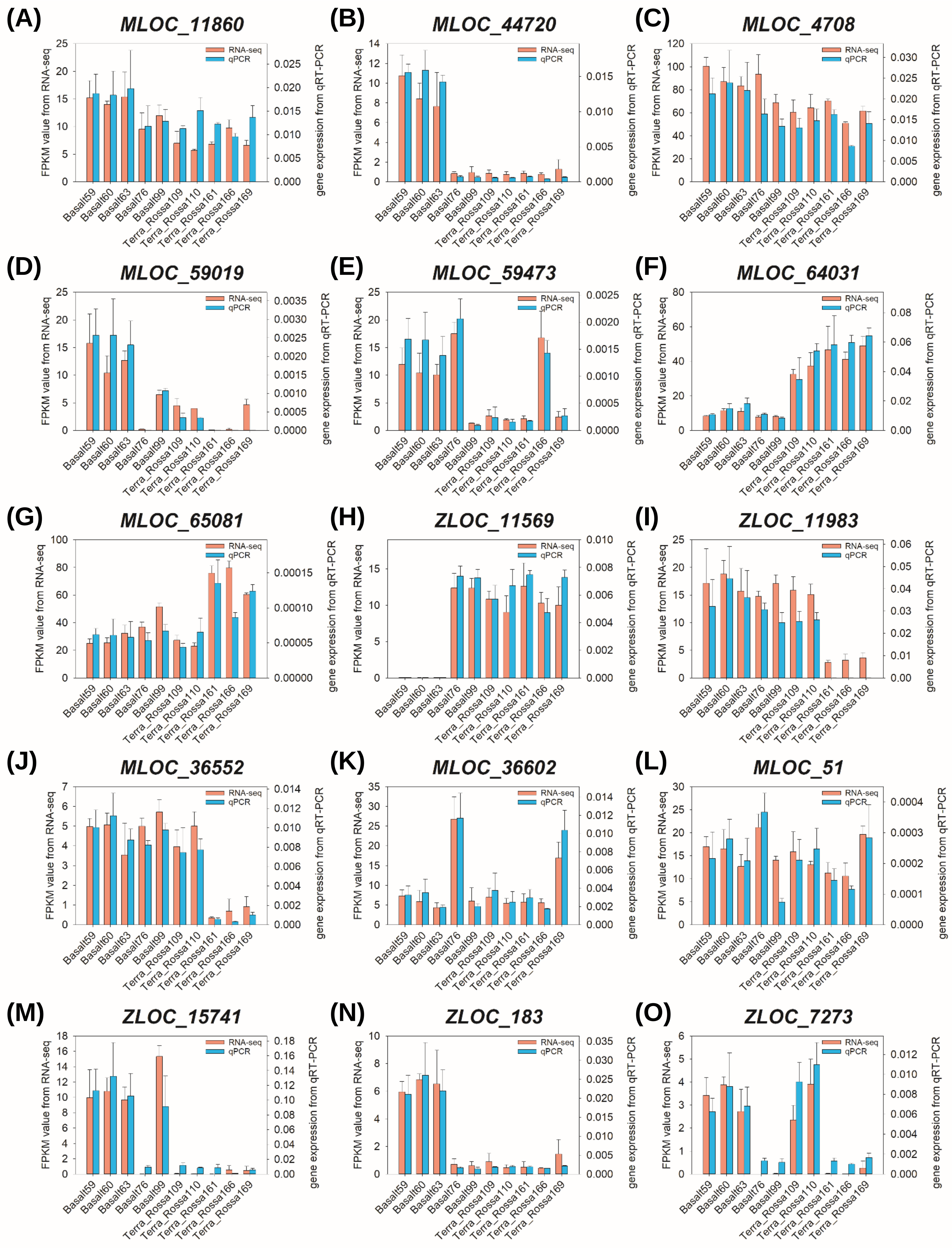

Figure S8. qRT-PCR verification of the gene expressions of 15 randomly selected genes in RNA-seq data. *HvActin* was used as reference gene in qRT-PCR analysis. Three biological replicates were applied for the leaves of each accession (mean ± SD).

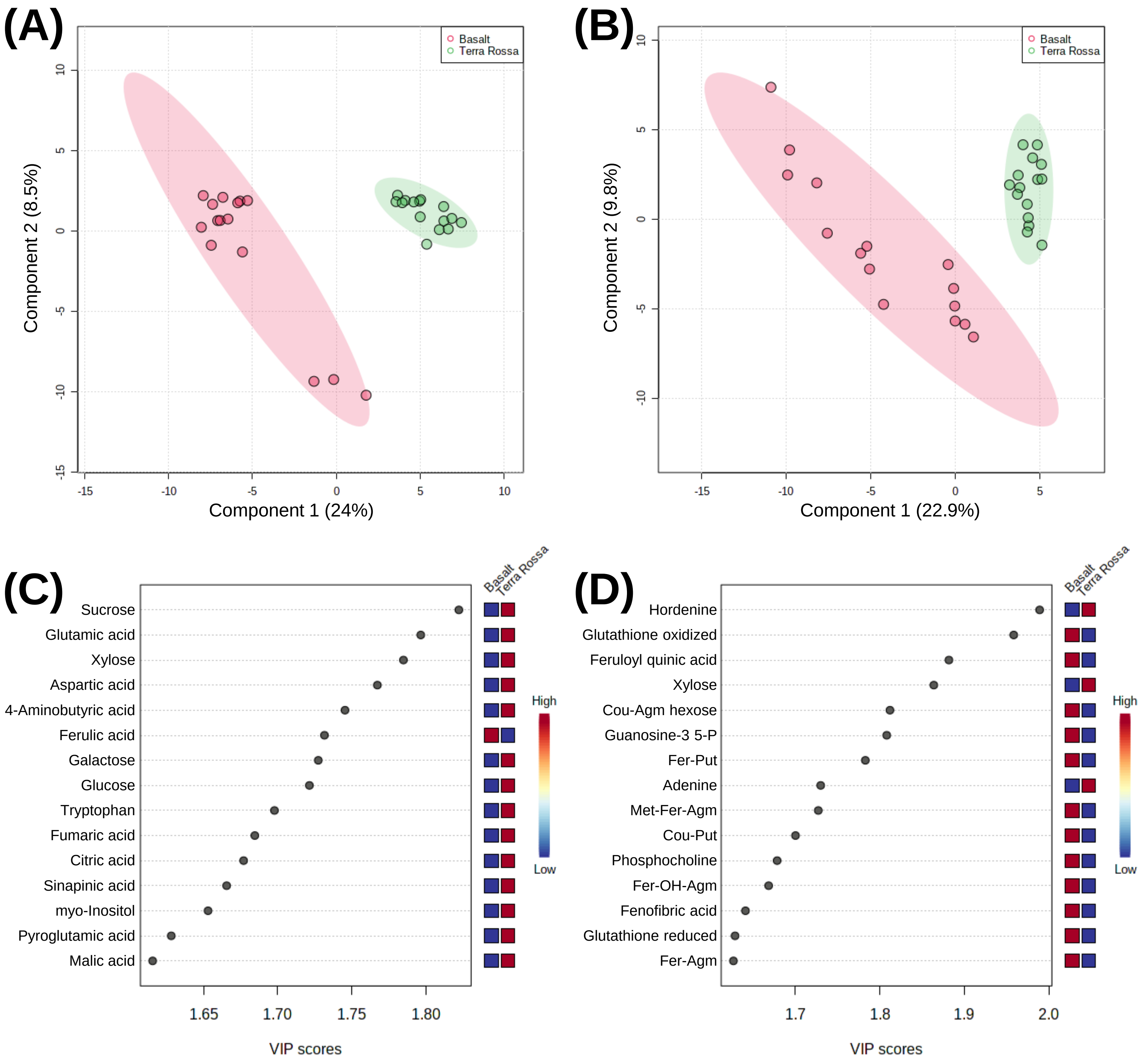

Figure S9. Principal component analysis (PCA) and VIP plot of metabolomic profiles of wild barley accessions from Basalt and Terra Rossa. A,B) PCA analysis of metabolite profiles in leaf (**A**) and root (**B**). **C,D)** VIP plot of metabolites in leaf (**C**) and root (**D**).

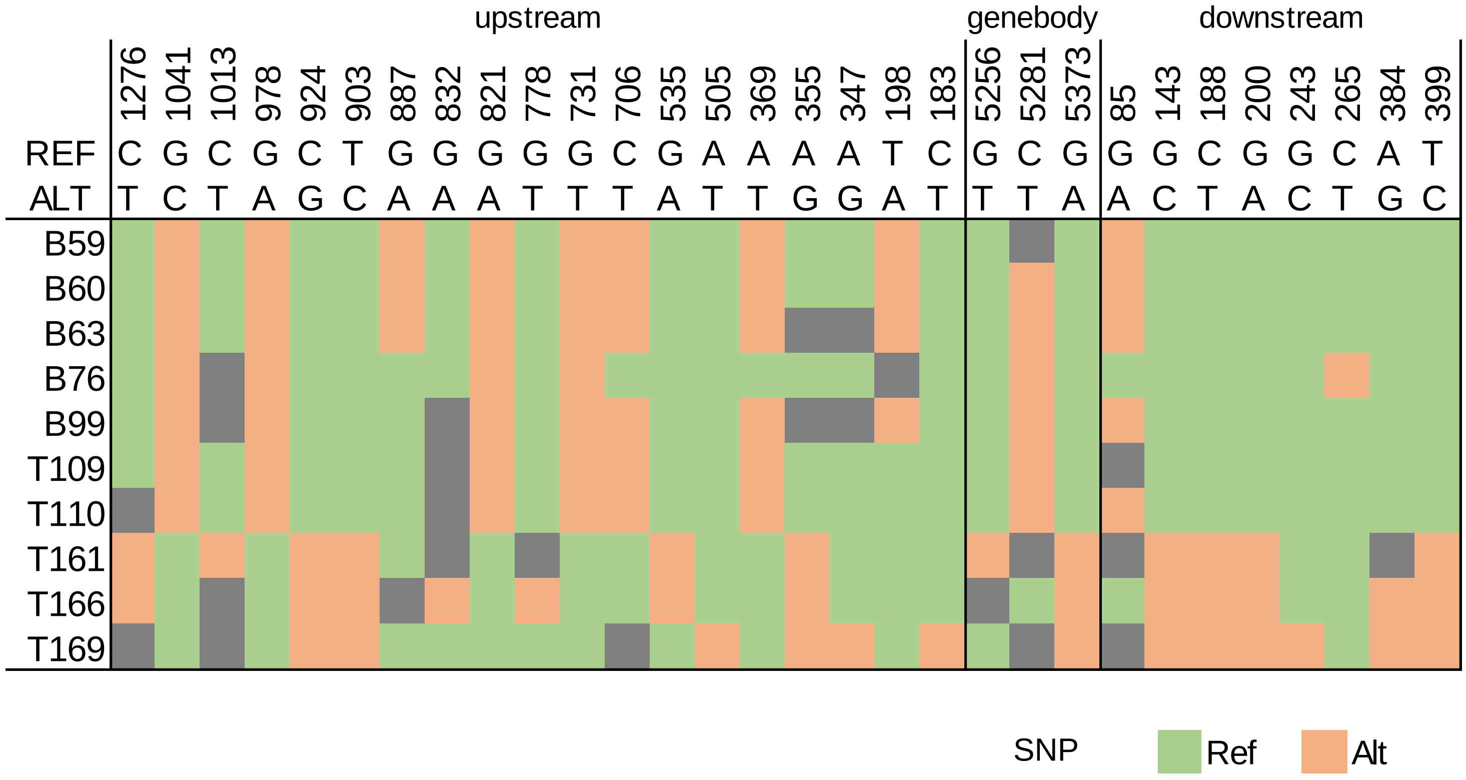

Figure S10. Distribution of SNPs in *ZLOC_11983* and its flanking region. Light green and orange represents reference locus and mutational locus aligned with ZQ320 reference genome.

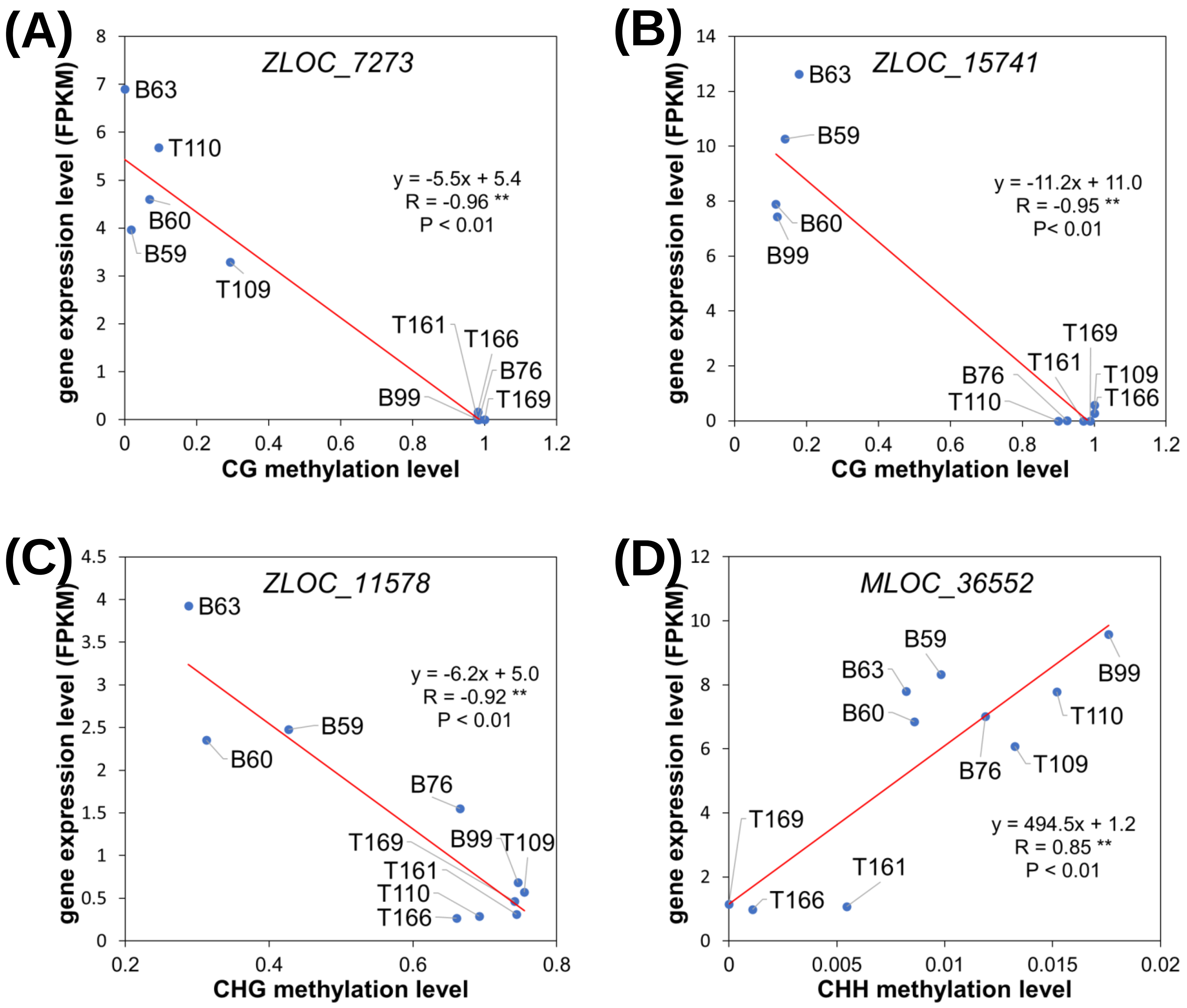

Figure S11. Correlation analysis between m5C level and transcriptional level. A) *ZLOC_7273*. **B)** *ZLOC_15741*. **C)** *ZLOC_11578*. **D)** *MLOC_36552*.

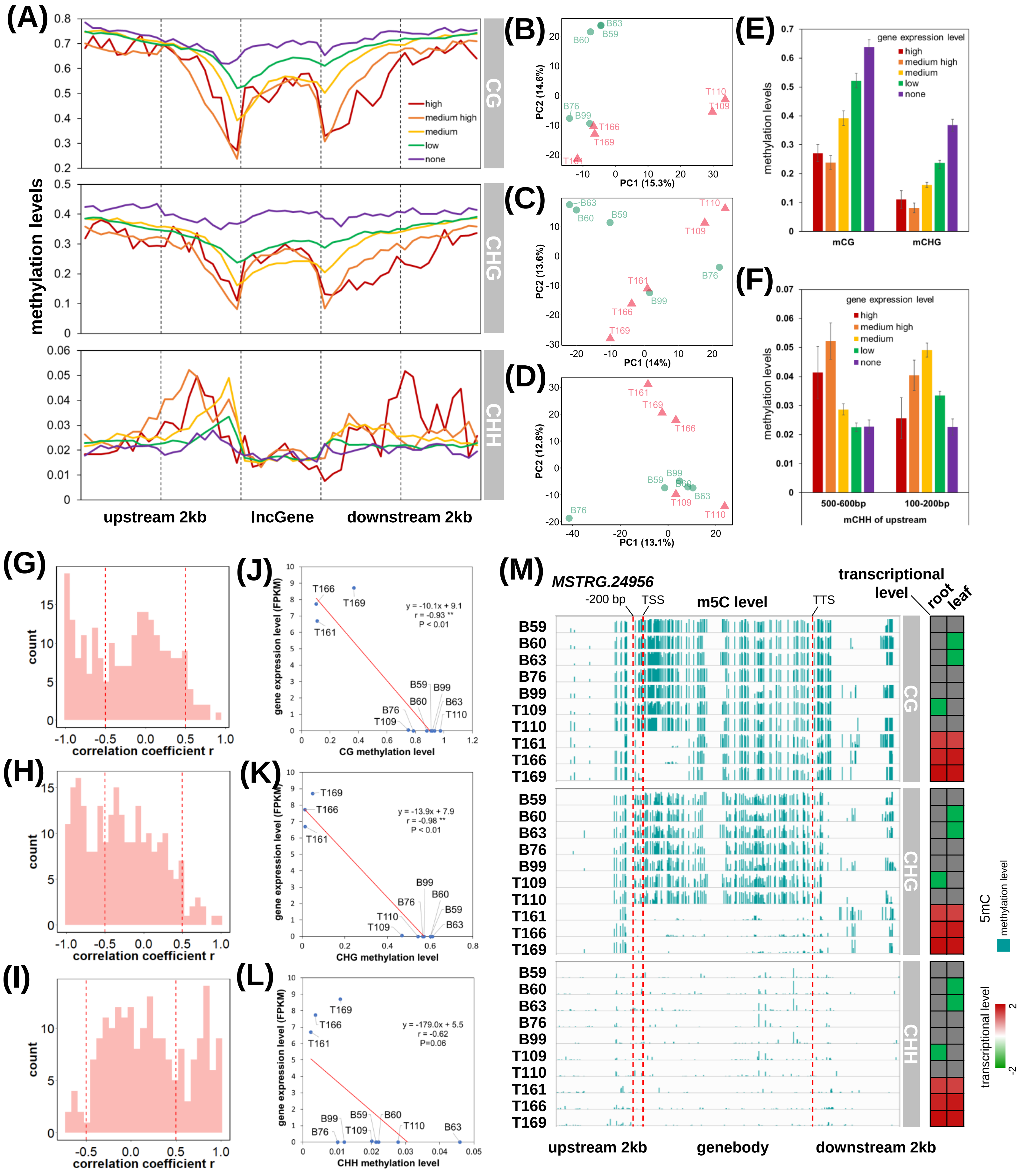

Figure S12. DNA methylation in lncRNA associated genomic region. A) Methylation levels within and flanking the genes partitioned by different expression levels. The gene expression was classified into five groups: high (FPKM>100), medium high (10<FPKM<100), medium (1<FPKM<10), low (0.1<FPKM<1) and none (FPKM value <0.1 was regarded as non-expressed). **B-D)** PCA analysis of DNA methylation in 200 bp upstream of lncRNA. **E)** The average CG and CHG methylation levels in the 100 bp upstream of TSS. **F)** The average CHH methylation levels in 100-200 and 500-600 bp upstream of TSS. **G-I)** distribution of pearson correlation coefficient between m5C level (**G**, mCG; **H**, mCHG; **I**, mCHH) and transcriptional level. **J-L)** correlation analysis between m5C level (**J**, mCG; **K**, mCHG; **L**, mCHH) and transcriptional level of *MSTRG.24956*. **M)** m5C level and transcriptional level of *MSTRG.24956.* B, Basalt; T, Terra Rossa.

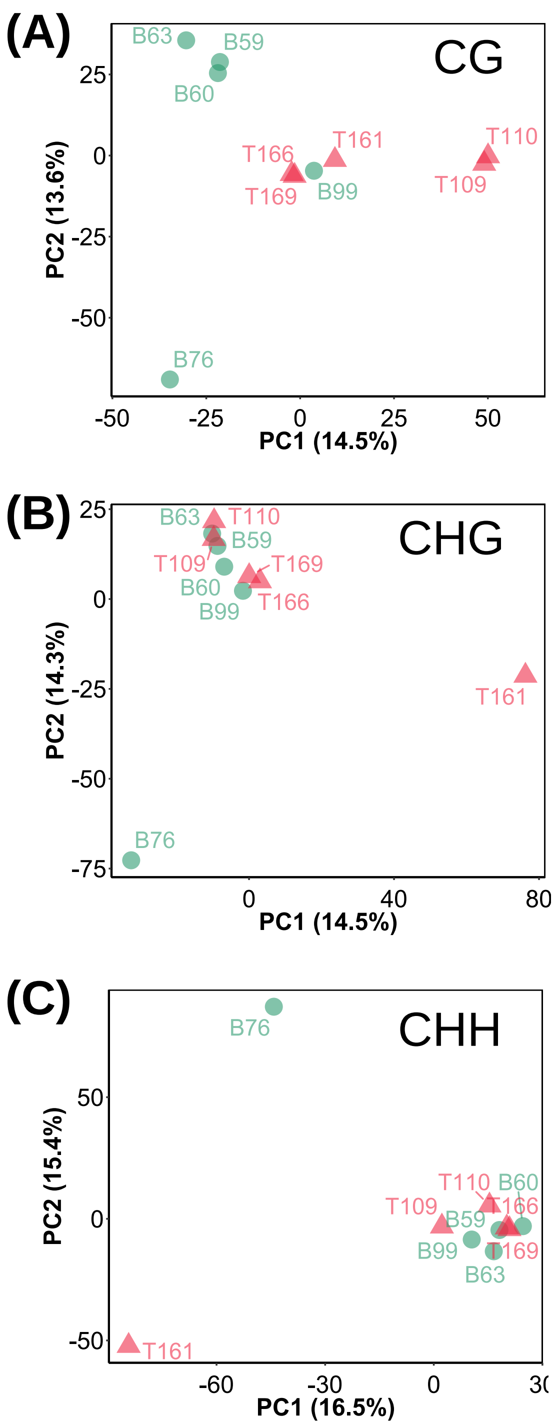

Figure S13. PCA analysis of DNA methylation in the 200 bp upstream of TSS in protein-coding genes. A) CG. **B)** CHG. **C)** CHH. B, Basalt; T, Terra Rossa.

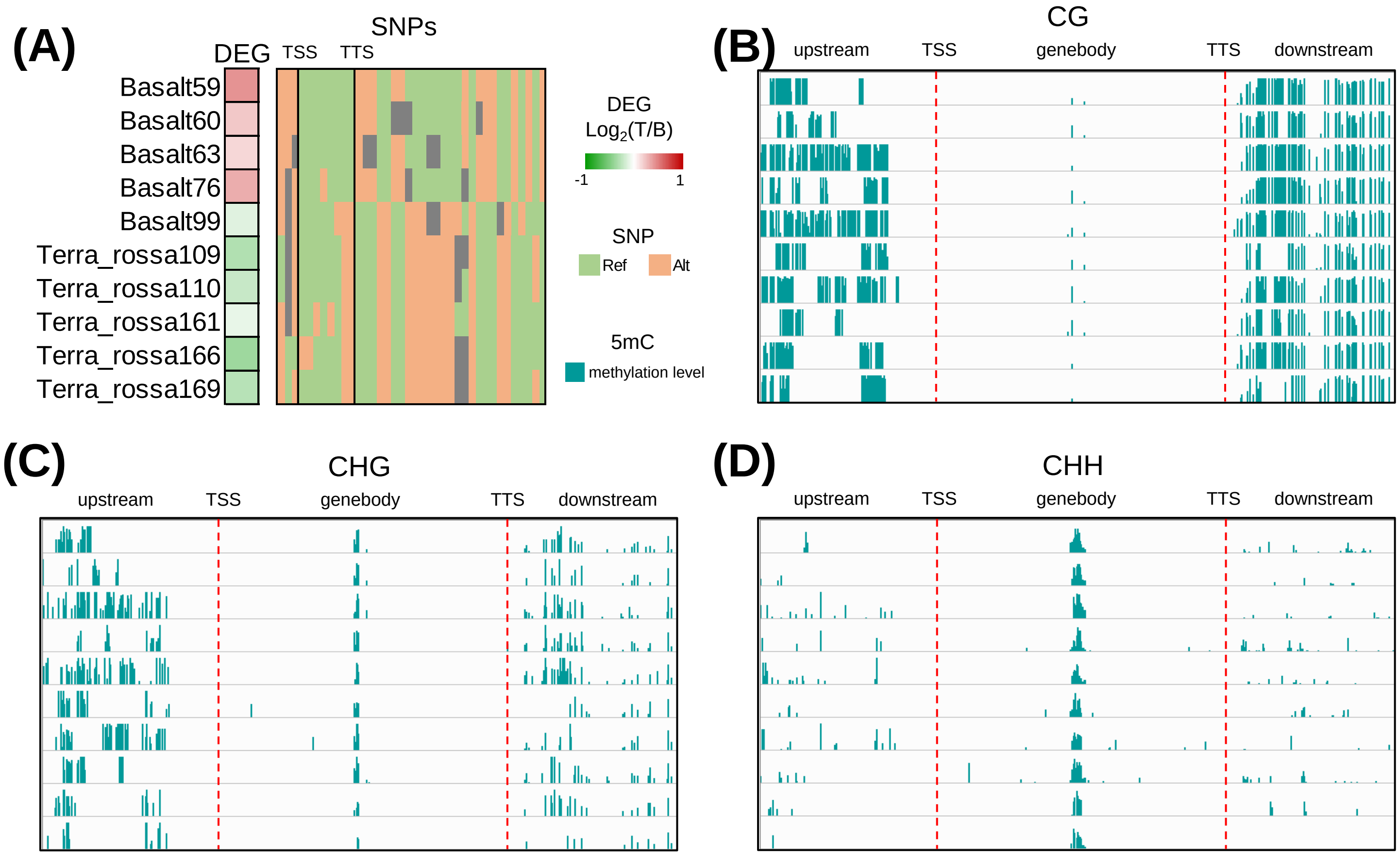

Figure S14. Genome, DNA methylation and gene expression of *C4H*. A) gene expression and SNPs. **B)** CG methylation. **C)** CHG methylation. **D)** CHH methylation.
